# *Arabidopsis* WRKY6 Transcription Factor Acts as a Positive Regulator of Abscisic Acid Signaling during Seed Germination and Early Seedling Development

**DOI:** 10.1371/journal.pgen.1005833

**Published:** 2016-02-01

**Authors:** Yun Huang, Cui-Zhu Feng, Qing Ye, Wei-Hua Wu, Yi-Fang Chen

**Affiliations:** State Key Laboratory of Plant Physiology and Biochemistry, College of Biological Sciences, China Agricultural University, National Plant Gene Research Centre (Beijing), Beijing, China; University of California Riverside, UNITED STATES

## Abstract

The phytohormone abscisic acid (ABA) plays important roles during seed germination and early seedling development. Here, we characterized the function of the *Arabidopsis* WRKY6 transcription factor in ABA signaling. The transcript of *WRKY6* was repressed during seed germination and early seedling development, and induced by exogenous ABA. The *wrky6-1* and *wrky6-2* mutants were ABA insensitive, whereas *WRKY6*-overexpressing lines showed ABA-hypersensitive phenotypes during seed germination and early seedling development. The expression of *RAV1* was suppressed in the *WRKY6*-overexpressing lines and elevated in the *wrky6* mutants, and the expression of *ABI3*, *ABI4*, and *ABI5*, which was directly down-regulated by RAV1, was enhanced in the *WRKY6*-overexpressing lines and repressed in the *wrky6* mutants. Electrophoretic mobility shift and chromatin immunoprecipitation assays showed that WRKY6 could bind to the *RAV1* promoter *in vitro* and *in vivo*. Overexpression of *RAV1* in *WRKY6*-overexpressing lines abolished their ABA-hypersensitive phenotypes, and the *rav1 wrky6-2* double mutant showed an ABA-hypersensitive phenotype, similar to *rav1* mutant. Together, the results demonstrated that the *Arabidopsis* WRKY6 transcription factor played important roles in ABA signaling by directly down-regulating *RAV1* expression.

## Author Summary

The WRKY6 protein is a WRKY transcription factor which plays important roles in plant pathogen defense, phosphate translocation, and arsenate resistance. This study demonstrated that the expression of *WRKY6* was obviously repressed during seed germination and significantly induced by exogenous ABA. In the presence of exogenous ABA, the two *wrky6* mutants showed ABA-insensitive phenotypes, whereas the *WRKY6*-overexpressing lines were hypersensitive to ABA. The WRKY6 transcription factor repressed *RAV1* expression and enhanced the expression of *ABI3*, *ABI4* and *ABI5*, which was down-regulated by RAV1. The WRKY6 protein could bind to the W-box motif within the *RAV1* promoter, indicating that WRKY6 directly regulated *RAV1* expression. Overexpression of *RAV1* abolished the ABA-sensitivity of *WRKY6*-overexpressing lines, and repression of *RAV1* impaired the ABA-insensitivity of *wrky6* mutants. Our results reveal the important roles of WRKY6 in ABA signaling during seed germination and early seedling development.

## Introduction

Abscisic acid (ABA) is a key phytohormone that plays important roles in plant responses to stresses and plant development [1–2]. ABA is accumulated in the developing embryo, and modulates seed development and storage product accumulation [1]. In addition, ABA prevents premature seed germination and controls seed dormancy to ensure that seeds germinate under favorable conditions [1]. After germination, ABA content declines rapidly [3–4], and exogenous ABA inhibits seed germination [5–6].

ABA functions through complex signaling networks, and some components of these networks are identified. The ABA receptors PYR/PYL/RCAR are identified in *Arabidopsis thaliana* [7–8]. Molecular genetics studies in *Arabidopsis* identify a number of genes involved in ABA signaling. The *snrk2*.*2 srnk2*.*3* double mutant shows strong ABA-insensitive phenotypes in seed germination and root growth inhibition, and the two protein kinases SnRK2.2 and SnRK2.3 are demonstrated to mediate a major part of ABA signaling during seed germination [9]. The *abi3*, *abi4*, and *abi5* mutants also show ABA-insensitive phenotypes during seed germination and early seedling development [10–12], and the *ABI3*, *ABI4*, and *ABI5* genes encode B3-type, APETALA2 domain and basic Leucine zipper (bZIP)-type transcription factors, respectively [10–13]. Three other bZIP-type transcription factors, AREB1/ABF2, AREB2/ABF4, and ABF3, are also involved in ABA signaling. During seed germination, none of the *areb1*, *areb2* and *abf3* mutants show ABA-sensitive phenotypes compared with wild-type plants, and during the vegetative growth stage, AREB1/ABF2, AREB2/ABF4, and ABF3 are key regulators of ABA signaling in response to osmotic stress [14–16].

The WRKY family is one of the largest transcription factor families in plants [17]. The WRKY proteins contain the conserved WRKY domain and zinc finger motif [18]. The conservation of the WRKY domain is mirrored by a remarkable conservation of the binding site, the W box (T)(T)TGAC(C/T) [18–19]. WRKY proteins act as repressors as well as activators by binding to their target genes’ promoters. Several WRKY transcription factors have been reported to be involved in the ABA signaling network. Three evolutionarily related WRKY transcription factors (AtWRKY18, AtWRKY40 and AtWRKY60) are negative regulators in ABA signaling, and AtWRKY40 directly represses the expression of *ABI4* and *ABI5* by binding to the promoters of *ABI4* and *ABI5* [20]. The knockout mutant of *AtWRKY63*, the *abo3* mutant, is hypersensitive to exogenous ABA during seed germination and the vegetative growth stage [21], and the *Arabidopsis wrky2* mutant has similar phenotypes to the *abo3* mutant except that AtWRKY2 has no effect on stomatal closure [22]. Recently, the AtWRKY41 protein is reported to control seed dormancy via direct regulation of *ABI3* expression [23], and AtWRKY8 functions in the TMV-cg defense response by mediating ABA and ethylene signaling [24].

In this study, we find that the *Arabidopsis* WRKY6 is a positive regulator in ABA signaling during seed germination and early seedling development. The knockout of *WRKY6* enhances plant ABA insensitivity during seed germination and early seeding growth, and *WRKY6*-overexpressing lines show ABA-hypersensitive phenotypes. The WRKY6 transcription factor represses *RAV1* expression and enhances the expression of *ABI3*, *ABI4* and *ABI5*, which are down-regulated by RAV1. The WRKY6 protein can bind to the W-box motif within the *RAV1* promoter, indicating that WRKY6 directly regulates *RAV1* expression. Overexpression of *RAV1* abolishes the ABA-sensitivity of *WRKY6*-overexpressing lines, and repression of *RAV1* impairs the ABA-insensitivity of *wrky6* mutants, demonstrating that *RAV1* is genetically epistatic to *WRKY6*.

## Results

### Disruption of *WRKY6* reduces, and overexpression of *WRKY6* enhances, ABA sensitivity during seed germination and early seedling development

**Fig 1:**
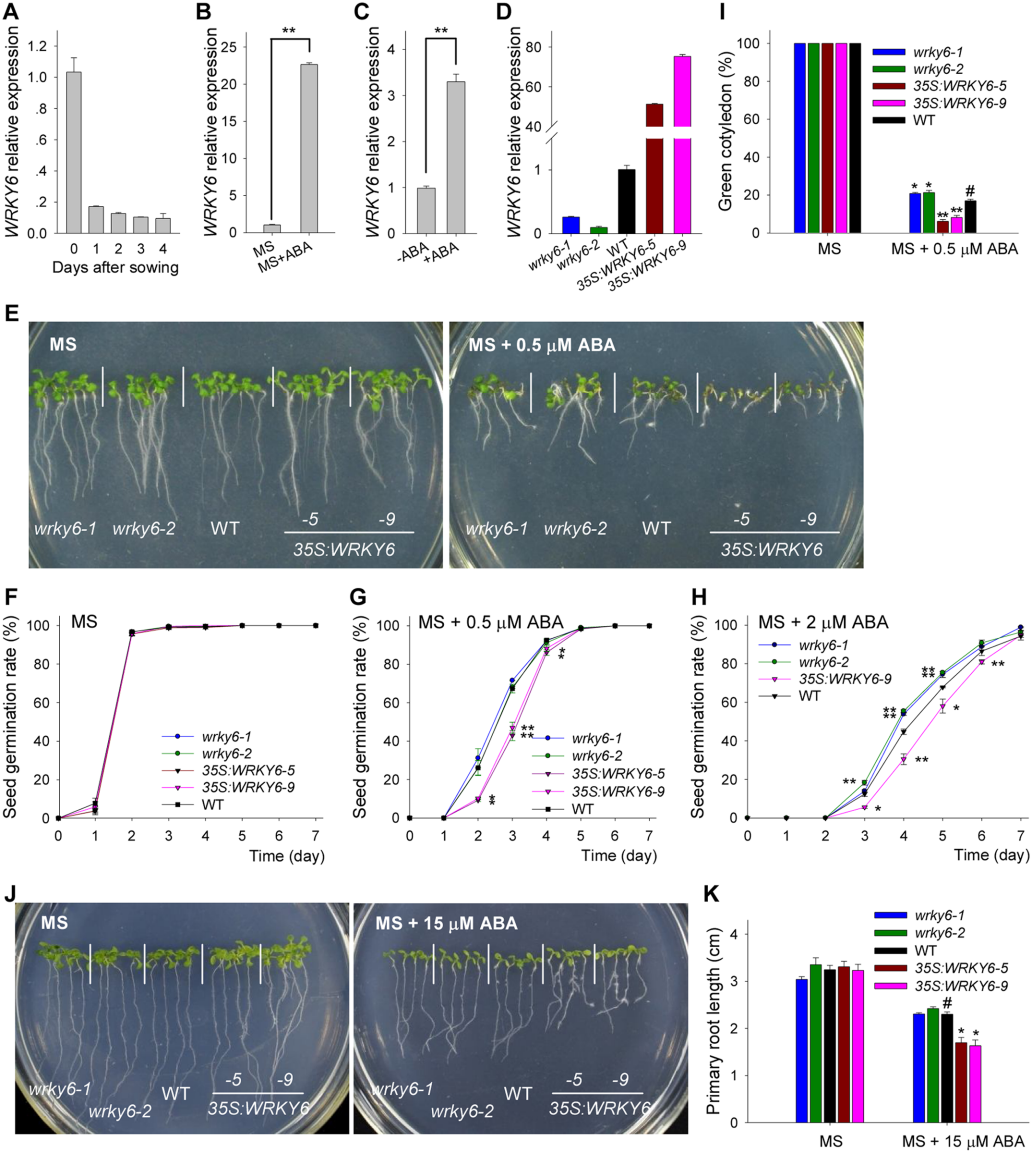
ABA-sensitivity of *wrky6* mutants and *WRKY6*-overexpressing lines. A, Expression of *WRKY6* was analyzed by qRT-PCR in wild-type plants (WT) during seed germination and early seedling development. The wild-type imbibed seeds were germinated and grown on MS medium, and then the plants were harvested at the indicated time. Data are shown as mean ± SE (n = 3). B, qRT-PCR analysis of *WRKY6* expression in response to exogenous ABA. Wild-type imbibed seeds were germinated on MS medium (MS) or MS medium with 0.5 M ABA (MSABA) for 1 d, and then the seeds were harvested. Data are shown as mean ± SE (n = 3). C, qRT-PCR analysis of *WRKY6* expression in 7-d-old wild-type seedlings treated with or without 100 M ABA for 3 h. Data are shown as mean ± SE (n = 3). D, Expression of *WRKY6* was analyzed by qRT-PCR in the *wrky6* mutants (*wrky6-1* and *wrky6-2*) and *WRKY6*-overexpressing lines (*35S*:*WRKY6-5* and *35S*:*WRKY6-9*). Data are shown as mean ± SE (n = 3). E, Phenotypic comparison. Imbibed seeds were transferred to MS or MS 0.5 μM ABA medium and grown for 10 d. F-H, Seed germination assay. Imbibed seeds were transferred to MS (F), MS medium containing 0.5 M ABA (G) or 2 M ABA (H), and then the seed germination rates were calculated at the indicated time. Data are shown as mean ± SE (n = 3). More than 300 seeds were measured in each replicate. I, Cotyledon-greening analysis. Imbibed seeds were transferred to MS or MS 0.5 μM ABA medium for 7 d before determining cotyledon-greening percentages. Data are shown as mean ± SE (n = 3). More than 300 seeds were measured in each replicate. J-K, Primary root length measurement with and without ABA addition. The 4-d-old seedlings were transferred to MS or MS 15 μM ABA medium for 7 d, and then the photos were taken and the primary root length was measured. Asterisks in G, H, I and K indicate statistically significant differences compared with wild-type plants: *, *P* 0.05; **, *P* 0.01. Wild-type plant (WT) was used as a control (#).

*Arabidopsis* WRKY6 (WRKY transcription factor 6, At1g62300) is a WRKY transcription factor [25] and, from public microarray data, we found that *WRKY6* expression was relatively high in dry seeds and reduced after imbibition. Then we examined the expression of *WRKY6* during seed germination and early seedling development. The transcript level of *WRKY6* was markedly repressed during seed germination (Fig 1A), indicating that WRKY6 may be involved in seed germination and early seedling development. When germinated and grown on Murashige and Skoog (MS) medium containing 0.5 μM ABA (MS+ABA), *WRKY6* expression was significantly induced (Fig 1B). The transcript level of *WRKY6* was further tested in seedlings treated with exogenous ABA. The 7-d-old wild-type seedlings were transferred to MS solution with or without 100 μM ABA for 3 h, and then harvested for qRT-PCR assay. The qRT-PCR results showed that the transcript level of *WRKY6* was significantly induced by exogenous ABA (Fig 1C).

*WRKY6*-overexpressing lines and *wrky6* mutants were used to study the physiological function of WRKY6 in seed germination. The *WRKY6*-overexpressing lines (*35S*:*WRKY6-5* and *35S*:*WRKY6-9*) and the *wrky6-1* mutant were provided by Dr. Somissich [26]. A *WRKY6* T-DNA insertion line (Salk_012997), named *wrky6-2*, was ordered from the ABRC (Arabidopsis Biological Resource Center). The qRT-PCR results showed that *WRKY6* expression was significantly repressed in the *wrky6-1* and *wrky6-2* mutants, and elevated in *35S*:*WRKY6-5* and *35S*:*WRKY6-9*, compared with wild-type plants (Fig 1D). When germinated and grown on MS medium, all plants showed no obvious difference in their phenotypes (Fig 1E, left panel). When grown on MS medium containing 0.5 μM ABA (MS + 0.5 μM ABA), the *wrky6-1* and *wrky6-2* mutants were more insensitive to ABA than wild-type plants, whereas *35S*:*WRKY6-5* and *35S*:*WRKY6-9* showed ABA hyper-sensitive phenotypes (Fig 1E, right panel). When grown on MS medium containing 0.5 μM ABA, the *wrky6-1* and *wrky6-2* mutants were less ABA insensitive than the *abi4* and *abi5* mutants (S1 Fig). Seed germination was further tested, and in the absence of ABA (MS), the seed germination percentages of different genotypes were similar (Fig 1F). When germinated and grown on MS medium containing 0.5 μM ABA (MS + 0.5 μM ABA), the two *WRKY6*-overexpressing lines (*35S*:*WRKY6-5* and *35S*:*WRKY6-9*) showed significantly reduced seed germination percentages, and the seed germination percentages of the two *wrky6* mutants were similar to wild-type plants (Fig 1G). When germinated and grown on MS medium containing 2 μM ABA (MS + 2 μM ABA), the two *WRKY6* mutants (*wrky6-1* and *wrky6-2*) showed significantly increased seed germination percentages compared with wild-type plants, and the *WRKY6*-overexpressing line (*35S*:*WRKY6-9*) showed reduced seed germination percentage relative to wild-type plants (Fig 1H). The cotyledon-greening percentages were also measured, and in the absence of ABA (MS), they were similar among different genotypes (Fig 1I). When germinated and grown on MS medium containing 0.5 μM ABA (MS + 0.5 μM ABA), the *WRKY6*-overexpressing lines (*35S*:*WRKY6-5* and *35S*:*WRKY6-9*) had lower, whereas the *wrky6* mutants (*wrky6-1* and *wrky6-2*) had higher, cotyledon-greening percentages than wild-type plants (Fig 1I).

To further test whether WRKY6 was involved in ABA mediated root growth inhibition, the 4-d-old *wrky6* mutants, *WRKY6*-overexpressing lines and wild-type seedlings were transferred to MS medium with or without 15 μM ABA for 7 d. When grown on MS medium, the primary root length was similar among different genotypes (Fig 1J and 1K). When grown on MS medium containing 15 μM ABA, the *WRKY6*-overexpressing lines (*35S*:*WRKY6-5* and *35S*:*WRKY6-9*) showed shorter primary root compared with wild-type seedlings, and the primary root lengths of *wrky6-1* and *wrky6-2* were similar to that of wild-type plants (Fig 1J and 1K). Together, these data indicate that WRKY6 plays important roles in ABA signaling during seed germination and seedling development.

### Disruption and overexpression of *WRKY6* alter expression of a set of ABA-responsive genes

**Fig 2:**
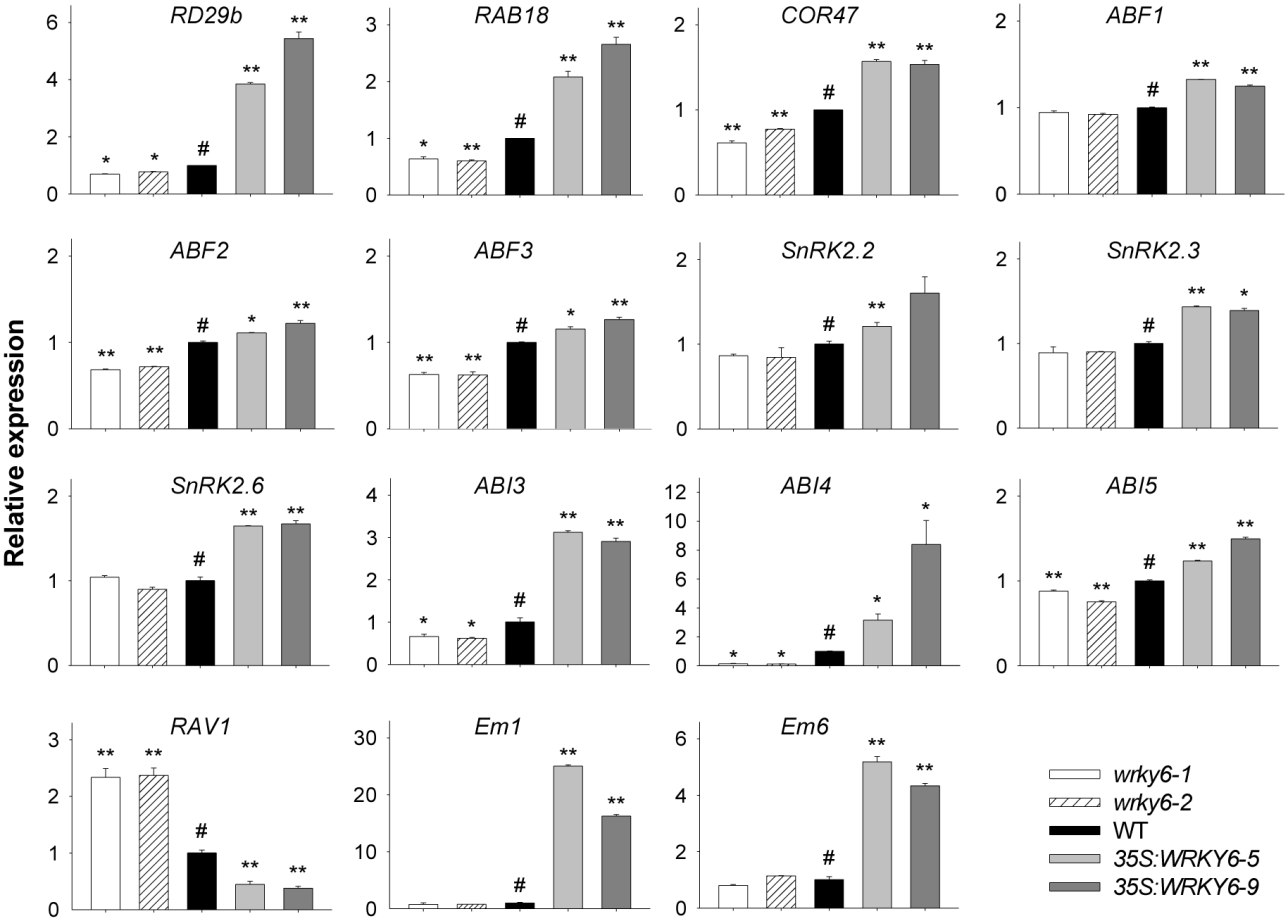
Expression of ABA-responsive genes in *wrky6* mutants and *WRKY6*-overexpressing lines. The imbibed seeds were germinated and grown on MS medium for 7 d, and then the seedlings were harvested for qRT-PCR. Data are shown as mean ± SE (n = 3). Asterisks indicate statistically significant differences compared with wild-type plants: *, *P* 0.05; **, *P* 0.01. Wild-type plant (WT) was used as a control (#).

As WRKY6 is a WRKY transcription factor involved in ABA signaling (Fig 1), the expression of ABA inducible genes, such as *RD29b*, *RAB18* and *COR47*, was tested in the *WRKY6*-overexpressing lines and *wrky6* mutants. The transcript levels of *RD29b*, *RAB18*, and *COR47* were elevated in the *WRKY6*-overexpressing lines and repressed in the *wrky6* mutants (Fig 2). Then the expression of the following ABA-responsive genes was tested: *ABFs* (*ABF1*, *ABF2/AREB1* and *ABF3*) [27], *SnRK2s* [28–29], *ABI3* [10], *ABI4* [11], *ABI5* [12], *RAV1* [30], *Em1* [31] and *Em6* [31]. The qRT-PCR results showed that the transcript levels of these ABA-responsive genes were elevated in the *WRKY6*-overexpressing lines, and the expression of most of these genes was repressed in the *wrky6* mutants (Fig 2). It is notable that the expression of *RAV1* was significantly repressed in the *WRKY6*-overexpressing lines and upregulated in the *wrky6-1* and *wrky6-2* mutants (Fig 2). It is also notable that the expression of *ABI3* and *ABI4* was elevated in the *WRKY6*-overexpressing lines and suppressed in the *wrky6* mutants (Fig 2).

**Fig 3:**
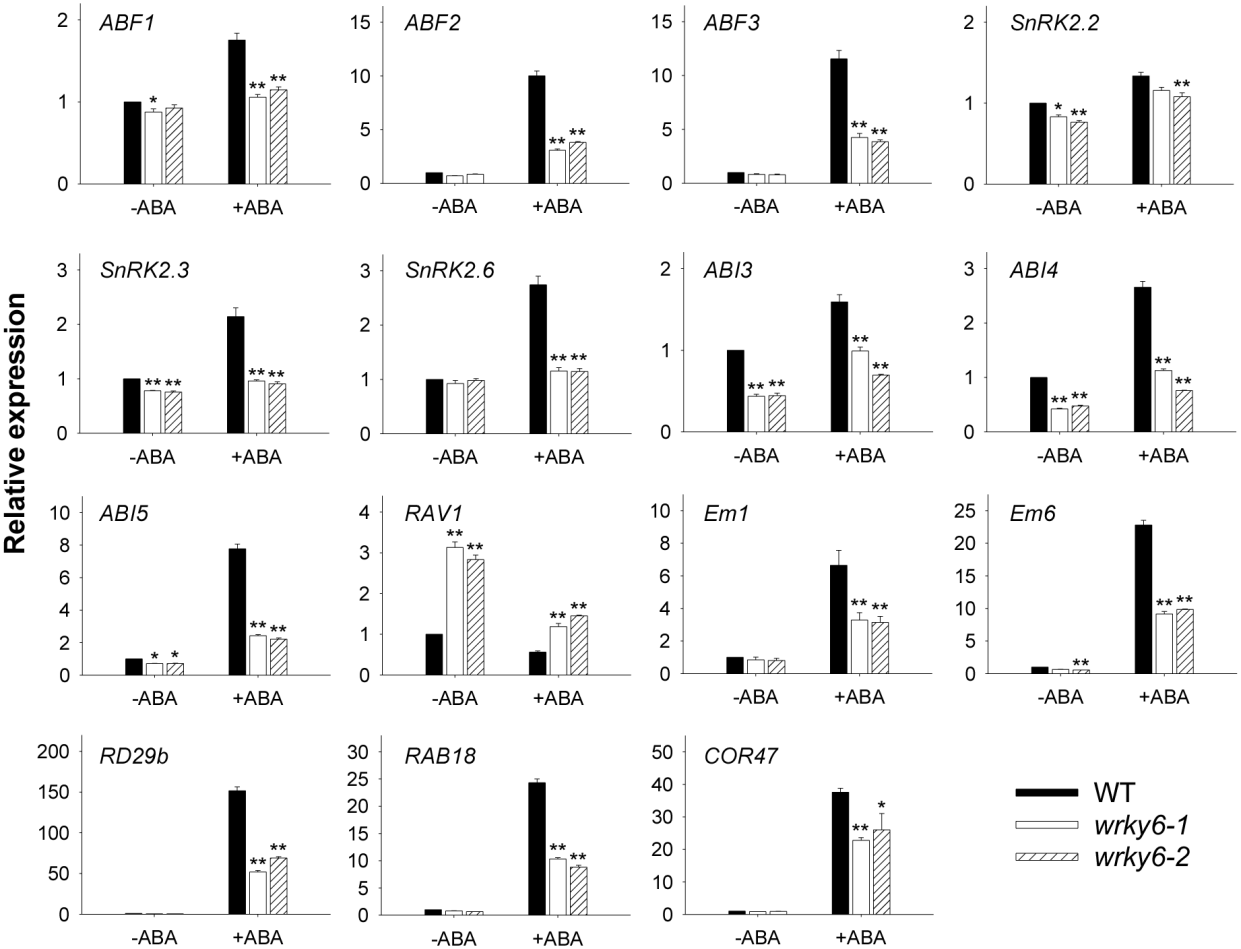
Expression of ABA-responsive genes in *wrky6* mutants and wild-type seedlings treated with exogenous ABA. The 7-d-old *wrky6* mutants and wild-type seedlings were transferred to MS solution with or without 100 μM ABA for 3 h, and then the seedlings were harvested for qRT-PCR. Data are shown as mean ± SE (n = 3). Asterisks indicate statistically significant differences compared with relevant wild-type plants (WT): *, *P* 0.05; **, *P* 0.01.

The expression of these ABA-responsive genes was also tested in the *wrky6* mutants and wild-type plants under exogenous ABA treatment. After the seedlings were treated with 100 μM ABA for 3 h, the expression of these genes, except *RAV1*, was induced in the wild-type seedlings, and this inducement by exogenous ABA was obviously repressed in the *wrky6-1* and *wrky6-2* mutants (Fig 3). The *RAV1* was repressed by exogenous ABA in the wild-type seedlings, and the transcript levels of *RAV1* in *wrky6-1* and *wrky6-2* mutants were much higher than that in wild-type seedlings with or without ABA treatment (Fig 3). These data demonstrate that disruption and overexpression of *WRKY6* alter the expression of the ABA-responsive genes. The expression of these genes was still ABA inducible in the *wrky6* mutants, indicating that besides WRKY6, there were other transcription factors regulating these genes expression.

### WRKY6 directly regulates *RAV1* expression

**Fig 4:**
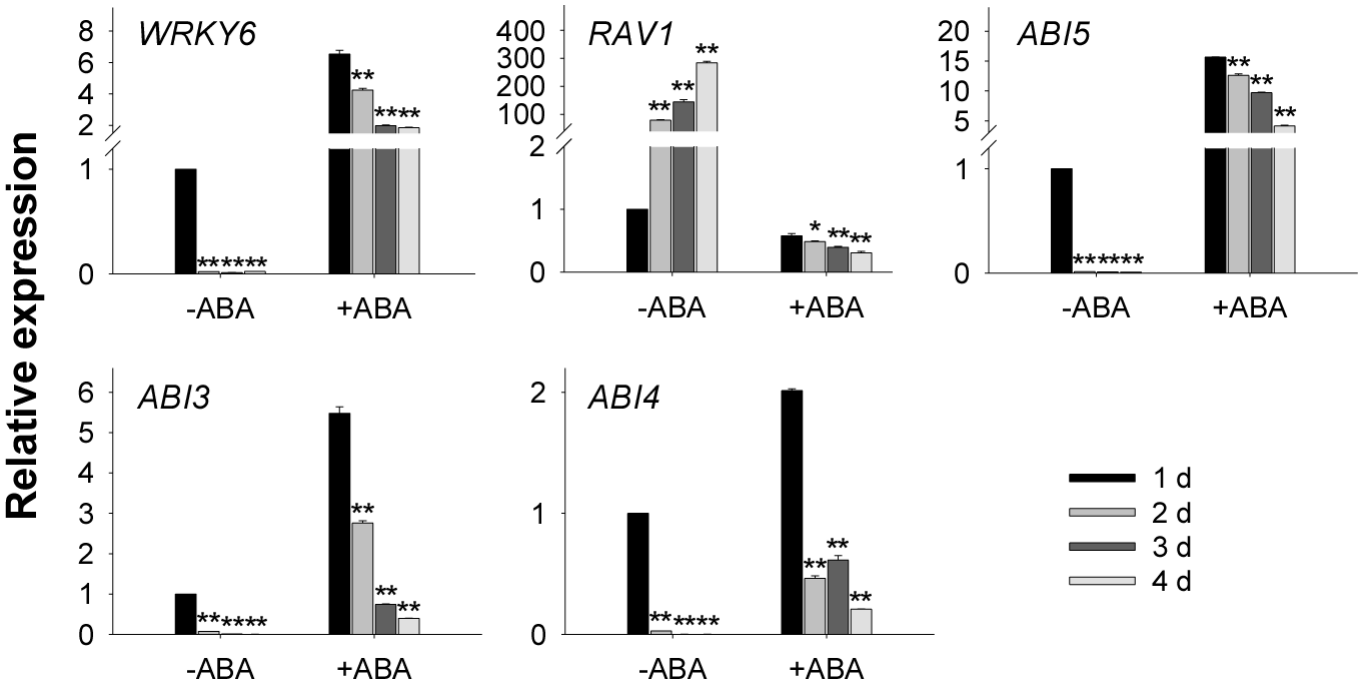
Expression of *WRKY6*, *RAV1* and *ABIs* in wild-type plants during seed germination and early seedling development. The imbibed wild-type seeds were transferred to the MS medium with or without 0.5 μM ABA, and then the plants were harvested at the indicated time for qRT-PCR. Data are shown as mean ± SE (n = 3). Asterisks indicate statistically significant differences compared with relevant wild-type plants (WT): *, *P* 0.05; **, *P* 0.01.

As our previous work showed that *Arabidopsis* RAV1 directly down-regulated the expression of *ABI3*, *ABI4*, and *ABI5* [30]—and the *RAV1* expression was lower, whereas the expression of *ABI3*, *ABI4* and *ABI5* was elevated in *WRKY6*-overexpressing lines (Figs 2 and 3)—we hypothesized that WRKY6 directly regulated *RAV1* expression. Then the expression of *ABI3*, *ABI4* and *ABI5* was further tested during the seed germination with or without exogenous ABA. During the seed germination, the transcript level of *WRKY6* was obviously repressed, and the *RAV1* expression was obviously induced (Fig 4). The transcript levels of *ABI3*, *ABI4* and *ABI5*, which directly down-regulated by RAV1, were obviously suppressed during seed germination (Fig 4). And the transcript levels of *WRKY6*, *ABI3*, *ABI4* and *ABI5* were obviously induced, and the *RAV1* expression was repressed, by exogenous ABA (Fig 4). These data imply that WRKY6 may directly regulate the *RAV1* expression.

**Fig 5:**
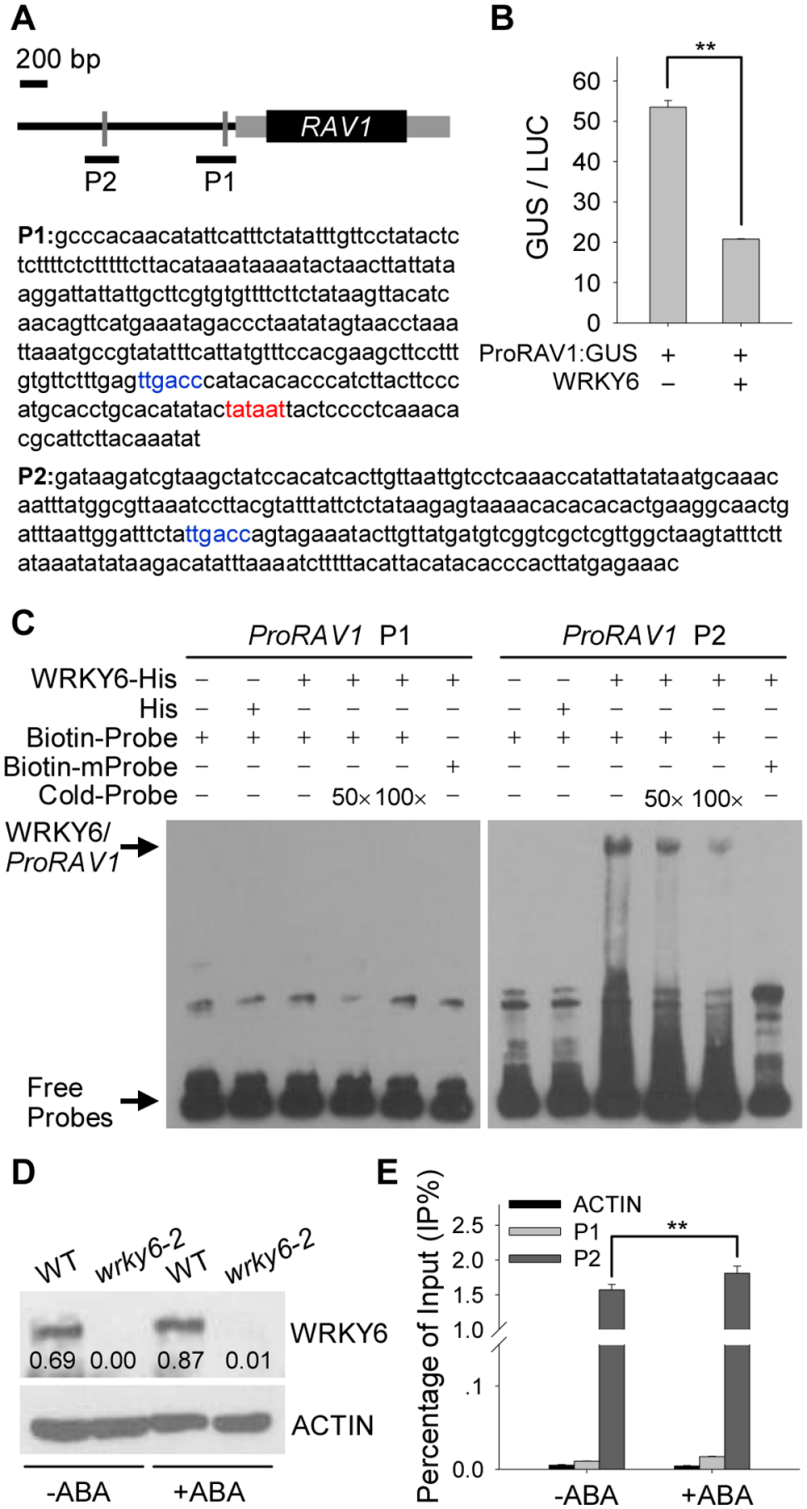
WRKY6 directly represses *RAV1* expression. A, Schematic representation of *RAV1* locus. *RAV1* putative promoter is indicated by black line showing relative positions of W-box motifs (gray lines), and transcribed sequence by black box (exon) and gray boxes (untranslated regions). Relative positions and sizes of different PCR-amplified fragments are indicated by black lines under the W-boxes. The sequence of W-box is shown in blue and the TATA box is shown in red. B, Transient overexpression of *WRKY6* fused to *ProRAV1*:*GUS* in *Nicotiana benthamiana* leaves. Data are shown as mean ± SE (n = 4). Asterisks indicate statistically significant differences: **, *P* 0.01. C, EMSA of WRKY6 binding to *RAV1* promoter *in vitro*. Each biotin-labeled DNA probe was incubated with WRKY6-His protein. The mutation probes of P1 and P2 have the mutated W-box (TTGACC was replaced by TACGTC). D, Immunoblot analysis of WRKY6 protein. The 7-d-old *wrky6-2* mutant and wild-type seedlings were transferred to MS solution with or without 100 μM ABA for 3 h, and then the seedlings were harvested for immunoblot analysis using anti-WRKY6 antibody. The relative band intensities of WRKY6, normalized relative to the intensity with the value of ACTIN (as 100%), are indicated by numbers below the bands. E, ChIP-qPCR assay of WRKY6 binding to *RAV1* promoter *in vivo*. The 7-d-old wild-type seedlings were transferred to MS solution with or without 100 μM ABA for 3 h, and then the seedlings were harvested for ChIP-qPCR assay using anti-WRKY6 antibody. Data are shown as mean ± SE (n = 3). Asterisks indicate statistically significant differences: **, *P* 0.01.

WRKY proteins act as regulators by binding to W-box(es) within their target genes promoters. First the *RAV1* promoter sequence was analyzed and the results showed that there were two W-box motifs within the *RAV1* promoter (Fig 5A). To further test the function of WRKY6 on regulation of *RAV1* expression, a transient expression experiment in tobacco leaves was performed—WRKY6 repressed *RAV1* promoter activity (Fig 5B).

Then an electrophoretic mobility shift assay (EMSA) was conducted to test whether WRKY6 bound to the *RAV1* promoter *in vitro*. The recombinant WRKY6-His protein and His protein alone were expressed in *Escherichia coli* and purified. The WRKY6-His fusion protein can bind to the P2 fragment of the *RAV1* promoter, and this binding was effectively reduced by adding increasing amounts of unlabeled competitor with the same P2 sequence (Fig 5C). When the W-box motif in the P2 fragment was mutated from TTGACC to TACGTC, the binding complex was not detected (Fig 5C). No super-shifted WRKY6-P1 complexes were detected in EMSA (Fig 5C). These data indicate that WRKY6 protein can bind to the P2 fragment of *RAV1* promoter *in vitro*.

Furthermore, a chromatin immunoprecipitation (ChIP) assay was conducted to determine whether WRKY6 bound to the *RAV1* promoter *in vivo*. The anti-WRKY6 antibody (AS111778; Agrisera) was tested in the *wrky6-2* mutant and wild-type seedlings, and the anti-WRKY6 antibody can specifically recognize the WRKY6 protein (Fig 5D). For the *WRKY6* expression was induced by exogenous ABA (Fig 1C), the protein level of WRKY6 was also tested under ABA treatment. After treated with 100 μM ABA for 3 h, the WRKY6 protein was elevated in the wild-type seedlings and still not detected in the *wrky6-2* mutant (Fig 5D). Then the ChIP assay was conducted with anti-WRKY6 antibody. The chromatin immunoprecipitated with the anti-WRKY6 antibody was enriched in the P2 fragment of the *RAV1* promoter, and the enrichment was enhanced under ABA treatment (+ABA) (Fig 5E). In contrast, fragments from the P1 fragment of the *RAV1* promoter and the exon region of the *Actin* gene (ACTIN) did not show any detectable binding by WRKY6 with or without ABA treatment (Fig 5E). These results demonstrate that WRKY6 directly regulates *RAV1* expression.

### Overexpression of *RAV1* abolishes ABA-sensitivity of the *WRKY6*-overexpressing line

**Fig 6:**
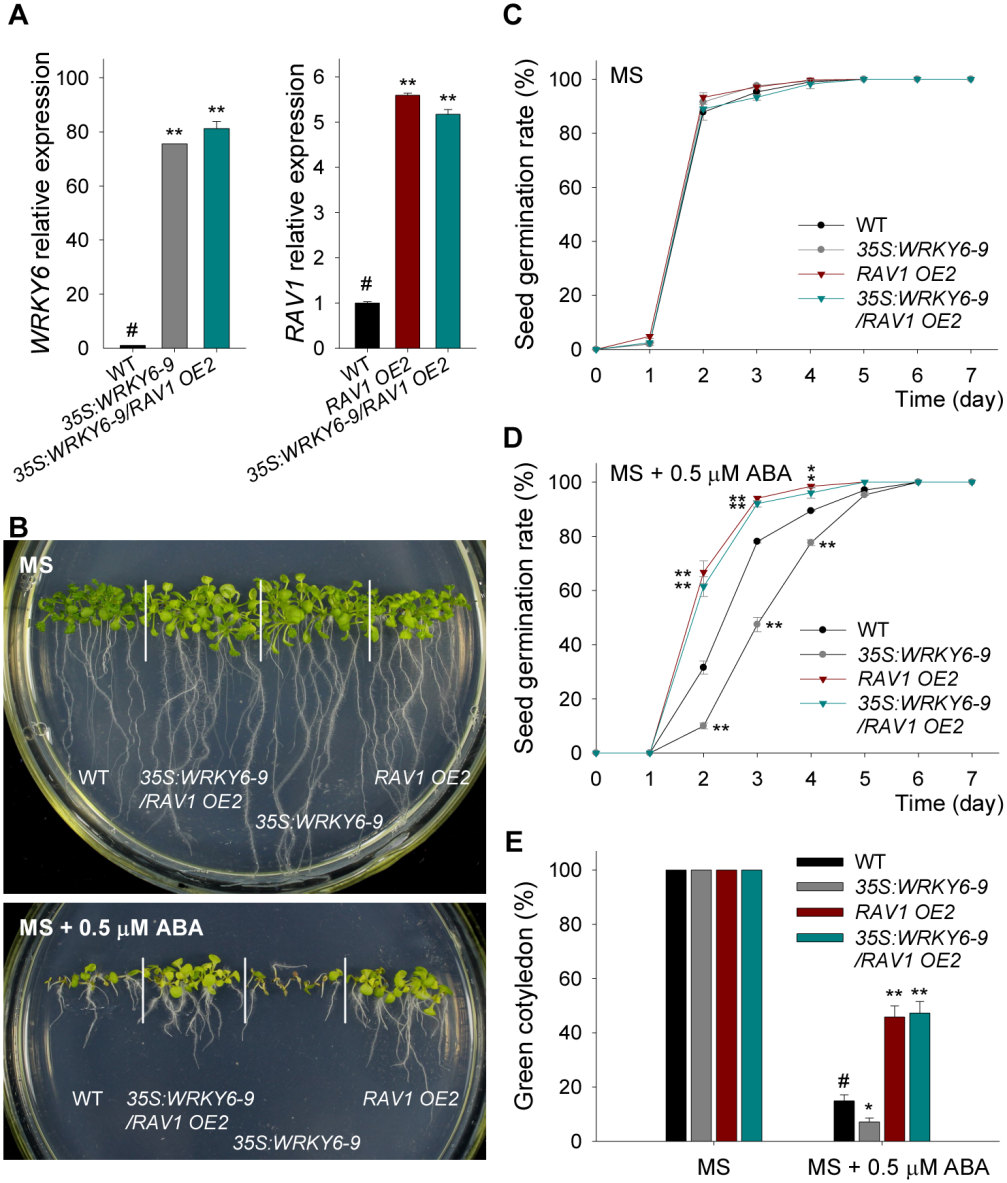
Overexpression of *RAV1* impairs the ABA-sensitive phenotypes of *WRKY6*-overexpressing line. A, The expression of *WRKY6* and *RAV1* was tested by qRT-PCR in *35S*:*WRKY6-9*, *RAV1 OE2*, *35S*:*WRKY6-9/RAV1 OE2* and wild-type plants (WT). Data are shown as mean ± SE (n = 3). B, Phenotypic comparison. Imbibed seeds were germinated and grown on MS medium (MS) or MS medium containing 0.5 μM ABA (MS + 0.5 μM ABA) for 10 d. C-D, Seed germination assay. Imbibed seeds were transferred to MS medium (C) or MS + 0.5 μM ABA medium (D), and then the seed germination rates were calculated at the indicated time. Data are shown as mean ± SE (n = 3). More than 300 seeds were measured in each replicate. E, Cotyledon-greening analysis. Imbibed seeds were germinated and grown on MS or MS + 0.5 μM ABA medium for 7 d before determining cotyledon-greening percentage. Data are shown as mean ± SE (n = 3). More than 300 seeds were measured in each replicate. Asterisks in D and E indicate statistically significant differences compared with wild-type plants: *, *P* < 0.05; **, *P* < 0.01. Wild-type plant (WT) was used as a control (#).

The *35S*:*WRKY6-9* was crossed with *RAV1*-overexpressing line (*RAV1 OE2*; [30]), and the *35S*:*WRKY6-9*/*RAV1 OE2* double overexpressing line was obtained (Fig 6A). When germinated and grown on MS medium, there were no obvious phenotype differences among all genotypes (Fig 6B, top panel), and their seed germination rates were similar (Fig 6C). In the presence of 0.5 μM ABA (MS + 0.5μM ABA), the *35S*:*WRKY6-9*/*RAV1 OE2* double overexpressing line displayed ABA-insensitive phenotypes, similar to *RAV1 OE2* (Fig 6B, bottom panel); and both *35S*:*WRKY6-9*/*RAV1 OE2* and *RAV1 OE2* had similar higher seed germination compared with wild-type plants, whereas the seed germination of *35S*:*WRKY6-9* was significantly reduced relative to wild-type (Fig 6D). The cotyledon-greening percentage was also measured. In the absence of ABA, the different genotypes had similar cotyledon-greening percentages (Fig 6E). In the presence of 0.5 μM ABA, both *35S*:*WRKY6-9*/*RAV1 OE2* and *RAV1 OE2* had higher, whereas the *35S*:*WRKY6-9* had lower, cotyledon-greening percentages than wild-type plants (Fig 6E).

**Fig 7:**
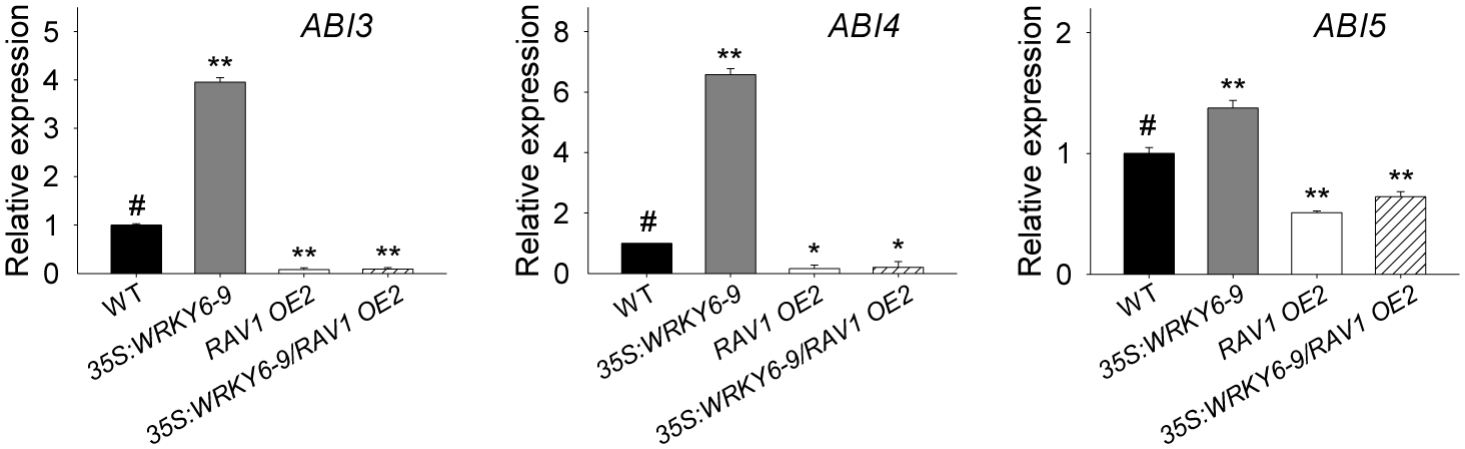
Expression of *ABI3*, *ABI4* and *ABI5* in *35S*:*WRKY6-9*, *RAV1 OE2*, *35S*:*WRKY6-9/RAV1 OE2* and wild-type plants. The imbibed seeds were germinated and grown on MS medium for 5 d, and then the seedlings were harvested for qRT-PCR. Data are shown as mean ± SE (n = 3). Asterisks indicate statistically significant differences compared with wild-type plants: *, *P* 0.05; **, *P* 0.01. Wild-type plant (WT) was used as a control (#).

Expression of RAV1 target genes, *ABI3*, *ABI4* and *ABI5*, was also tested by qRT-PCR and all were elevated in the *WRKY6*-overexpressing line (*35S*:*WRKY6-9*), but repressed in the *35S*:*WRKY6-9*/*RAV1 OE2* lines, similar to *RAV1 OE2*, compared with wild-type plants (Fig 7). These data together with phenotype tests indicated that *RAV1* overexpression abolished the ABA-sensitivity of *WRKY6*-overexpressing line.

**Fig 8:**
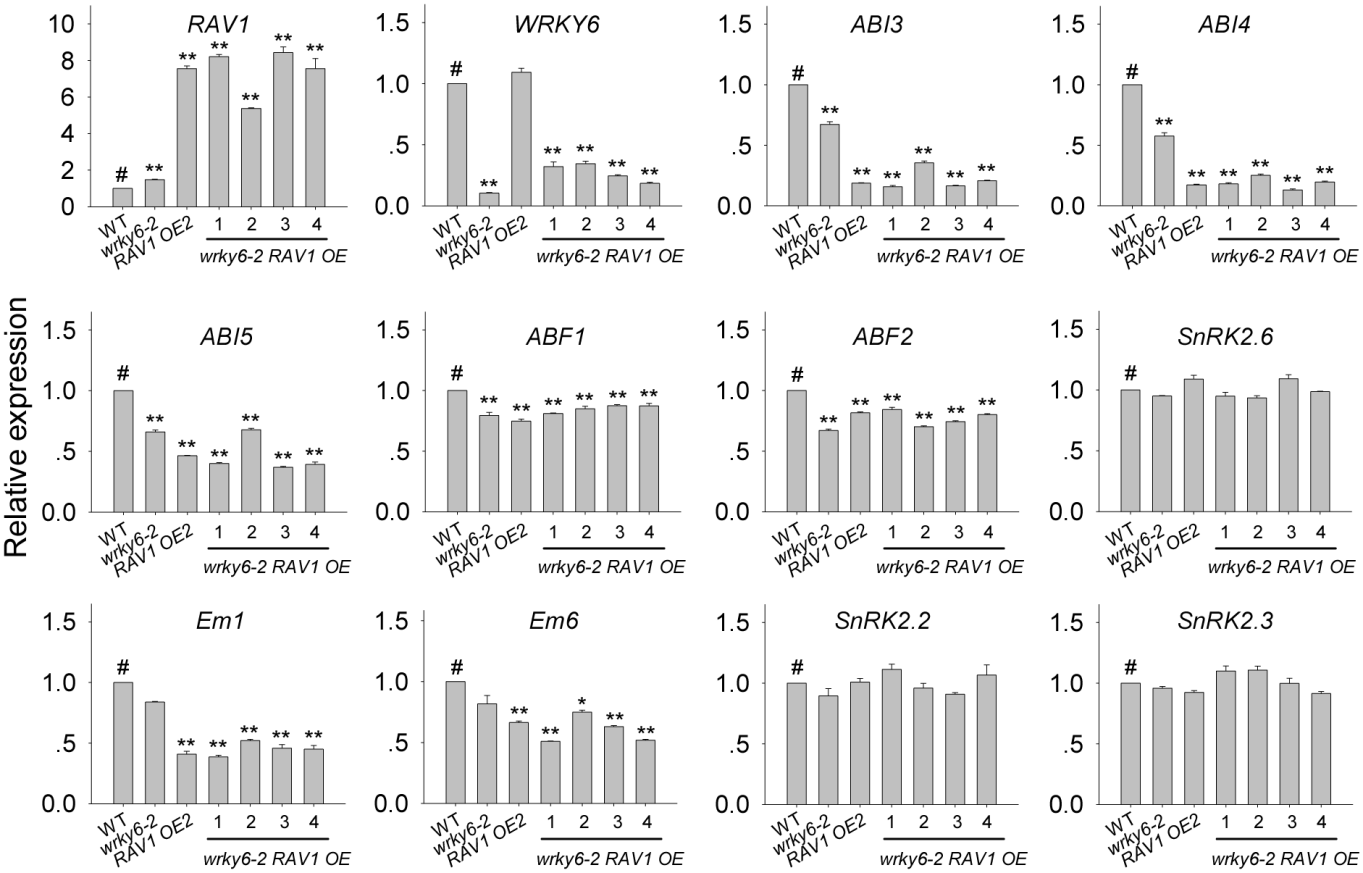
Expression of *ABFs*, *Ems* and *SnRK2s* in the *wrky6-2* mutant, *RAV1 OE2*, *wrky6-2 RAV1 OE* transgenic lines and wild-type plants. The genes expression was tested by qRT-PCR in the *wrky6-2* mutant, *RAV1 OE2*, *wrky6-2 RAV1 OE* transgenic lines (T1) and wild-type plants (WT). Three technical replicates were performed. Asterisks indicate statistically significant differences compared with wild-type plants: *, *P* 0.05; **, *P* 0.01. Wild-type plant (WT) was used as a control (#).

We also introduced *Super*:*RAV1* [30] to the *wrky6-2* mutant, and got four *wrky6-2 RAV1OE* (T1) transgenic lines (Fig 8). The four *wrky6-2 RAV1OE* lines had the higher *RAV1* expression and much lower *WRKY6* expression than wild-type plants (Fig 8). The transcript levels of *ABI3*, *ABI4* and *ABI5* in *wrky6-2 RAV1 OE* lines were lower than those in wild-type plants, even lower than those in the *wrky6-2* mutant, similar to those in *RAV1 OE2* (Fig 8). The transcript levels of *Em1* and *Em6* in *wrky6-2 RAV1OE* lines were also lower than those in wild-type and *wrky6-2* mutant, similar to those in *RAV1 OE2* (Fig 8). These data indicate that overexpression of *RAV1* represses the expression of *ABI3*, *ABI4* and *ABI5*, and WRKY6 modulates the expression of *ABI3*, *ABI4* and *ABI5* through down-regulating the *RAV1* expression. The expression of *ABFs* and *SnRK2s* was also tested. The transcript levels of *ABF1* and *ABF2* in the *wrky6-2* mutant, *RAV1 OE2* and *wrky6-2 RAV1OE* lines were similar, and slightly lower than those in wild-type plants (Fig 8). And the transcript levels of *SnRK2s* were similar among *RAV1 OE2*, *wrky6-2 RAV1 OE* lines and wild-type plants (Fig 8).

### Disruption of *RAV1* abolishes ABA-insensitivity of the *wrky6* mutant

**Fig 9:**
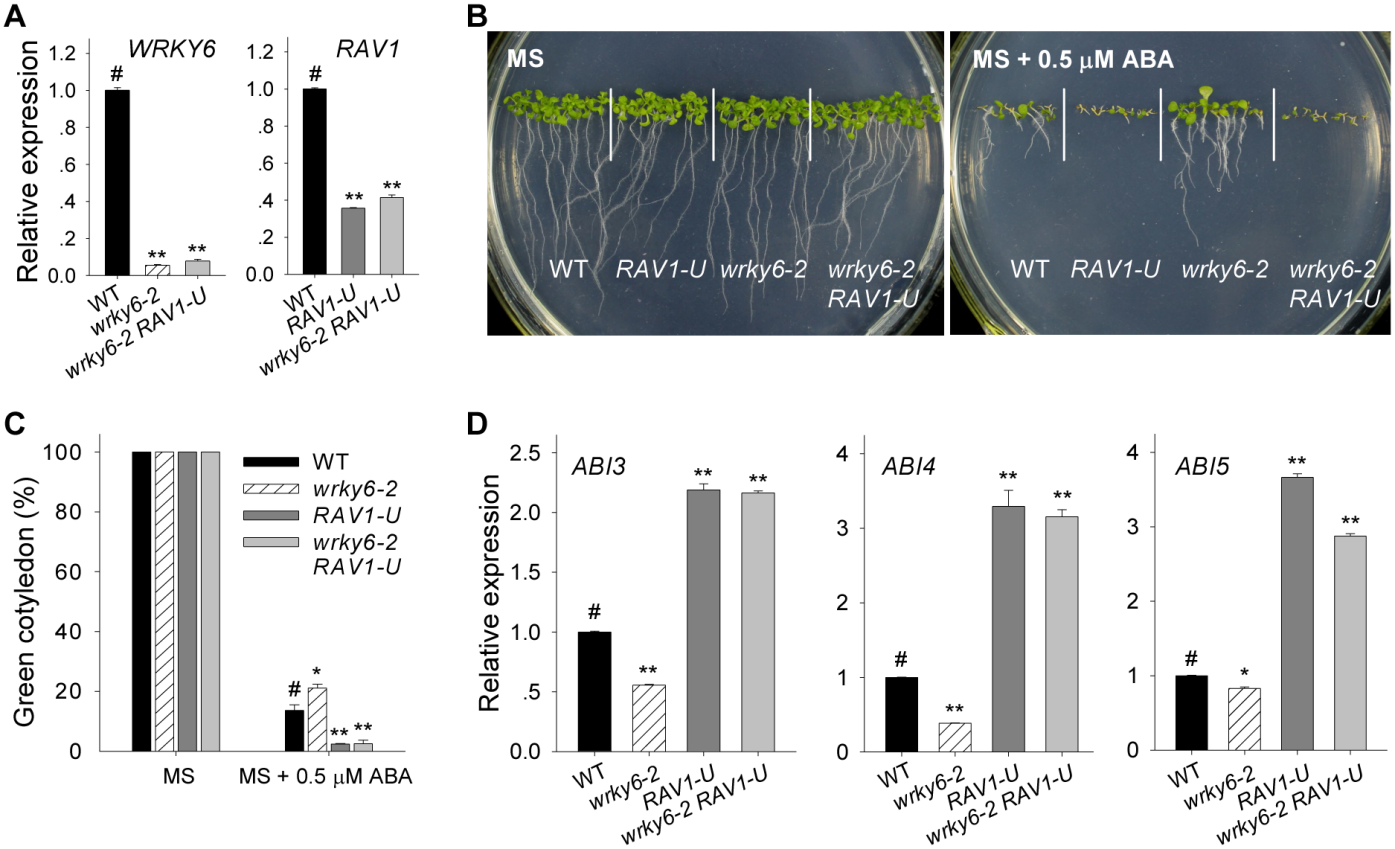
The ABA-insensitivity of the *wrky6* mutant is abolished by suppression of *RAV1*. A, The transcript levels of *WRKY6* and *RAV1* were tested by qRT-PCR. Data are shown as mean ± SE (n = 3). B, Phenotypic comparison. Imbibed seeds were transferred to MS medium (MS) or MS medium containing 0.5 μM ABA (MS + 0.5 μM ABA) for 10 d. C, Cotyledon-greening analysis. Imbibed seeds were transferred to MS or MS + 0.5 μM ABA medium for 7 d before determining cotyledon-greening percentage. Data are shown as mean ± SE (n = 3). More than 300 seeds were measured in each replicate. D, Expression of *ABI3*, *ABI4*, and *ABI5* was tested by qRT-PCR in the *wrky6-2* mutant, *RAV1-U*, *wrky6-2 RAV1-U* double mutant and wild-type plants. Each data represents the mean ± SE (n = 3). Asterisks indicate statistically significant differences compared with wild-type plants: *, *P* < 0.05; **, *P* < 0.01. Wild-type plants (WT) were used as a control (#).

The *RAV1*-underexpressing line (*RAV1-U*) is an antisense transgenic line, which has relatively lower *RAV1* expression [30, 32]. When grown on MS medium containing 0.5 μM ABA, *RAV1-U* shows ABA hyper-sensitive phenotypes [30]. The genetic relationship between *WRKY6* and *RAV1* was analyzed by crossing *wrky6-2* with *RAV1-U* to produce the *wrky6-2 RAV1-U* double mutant (Fig 9A). In the absence of ABA (MS), all lines showed similar phenotypes (Fig 9B, left panel). When germinated and grown on MS medium containing 0.5 μM ABA (MS + 0.5 μM ABA), the *wrky6-2* mutant displayed an ABA-insensitive phenotype, whereas the *wrky6-2 RAV1-U* double mutant showed an ABA-sensitive phenotype, similar to *RAV1-U* (Fig 9B, right panel). The cotyledon-greening percentages were also tested and, in the absence of ABA (MS), were similar for the different genotypes (Fig 9C). When germinated and grown on MS medium containing 0.5 μM ABA (MS + 0.5 μM ABA), the *wrky6-2 RAV1-U* double mutant (similar to *RAV1-U*) had a much lower, and the *wrky6-2* mutant had a higher, cotyledon-greening percentage than wild-type plants (Fig 9C).

Expression of *ABI3*, *ABI4* and *ABI5* was tested by qRT-PCR and showed clearly elevated transcript levels in the *wrky6-2 RAV1-U* double mutant, similar to that in *RAV1-U*, and repressed in the *wrky6-2* mutant (Fig 9D).

**Fig 10:**
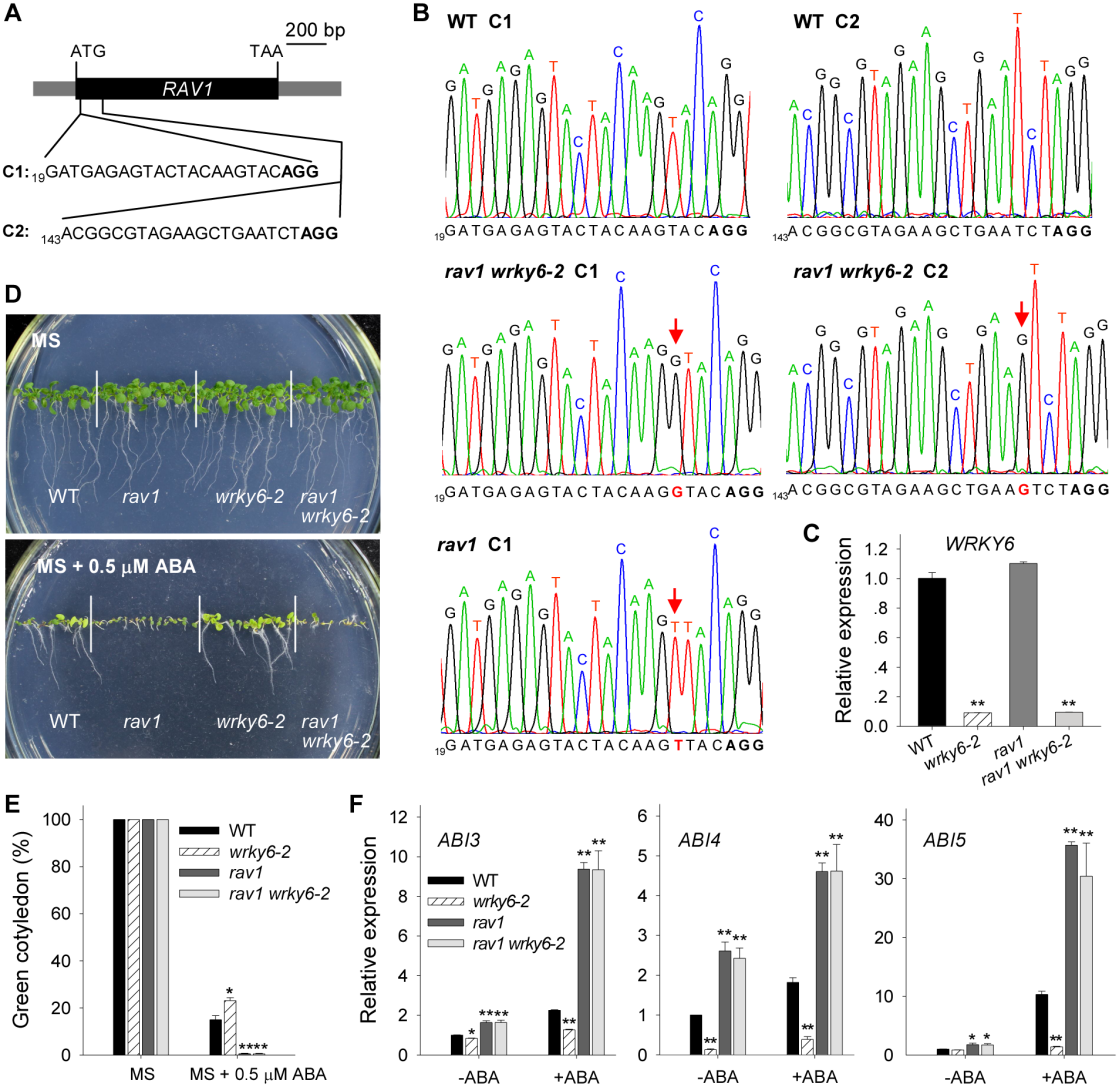
Disruption of *RAV1* abolishes the ABA-insensitivity of the *wrky6* mutant. A, Diagram of *RAV1* showing two target sites (C1 and C2) for CRISPR/Cas9 technology. PAM motifs are marked with bold letters. The coding sequence (CDS) and untranslated regions (UTR) of *RAV1* are indicated by black box and gray boxes, separately. B, The *rav1* mutant and *rav1 wrky6-2* double mutant were generated by CRISPR/Cas9 technology. The mutation in the *RAV1* gene is evaluated by sequencing, and the mutant sites in *RAV1* are indicated by red letters and arrows. C, qRT-PCR analysis of *WRKY6* expression in the *wrky6-2* mutant, *rav1* mutant, *rav1 wrky6-2* double mutant and wild-type plants (WT). Data are shown as mean ± SE (n = 3). D, Phenotypic comparison. Imbibed seeds were transferred to MS or MS + 0.5 μM ABA medium for 10 d. E, Cotyledon-greening analysis. Imbibed seeds were transferred to MS or MS + 0.5 μM ABA medium for 7 d before determining cotyledon-greening percentage. Data are shown as mean ± SE (n = 3). F, qRT-PCR analysis of *ABIs* in the *wrky6-2* mutant, *rav1* mutant, *rav1 wrky6-2* double mutant and wild-type plants (WT) treated with or without exogenous ABA treatment. The 7-d-old seedlings were transferred to MS solution with or without 100 μM ABA for 3 h, and then the seedlings were harvested for qRT-PCR. Data are shown as mean ± SE (n = 3). Asterisks indicate statistically significant differences compared with relevant wild-type plants (WT): *, *P* 0.05; **, *P* 0.01.

Further, we used the CRISPR/Cas9 technology to generate *rav1* mutant and *rav1 wrky6-2* double mutant. A pair of closely located sgRNA targets in the *RAV1* gene were selected (Fig 10A). The CRISPR construct was transformed into wild-type *Arabidopsis* and the *wrky6-2* mutant, and the homozygous *rav1* mutant and *rav1 wrky6-2* double mutant were obtained. The *rav1 wrky6-2* double mutant contained a nucleotide insertion in C1 and C2 sites, separately (Fig 10B), and the *rav1* mutant had a nucleotide insertion in C1 site (Fig 10B). These insertions lead to frameshift mutation. The qRT-PCR results showed that the transcript level of *WRKY6* was significantly repressed in the *rav1 wrky6-2* double mutant, similar to that in *wrky6-2* mutant (Fig 10C). These data indicate that we obtain the *rav1* mutant and *rav1 wrky6-2* double mutant.

When grown on MS medium containing 0.5 μM ABA, the *rav1 wrky6-2* double mutant showed ABA hyper-sensitive phenotypes, similar to the *rav1* mutant (Fig 10D), and both *rav1* mutant and *rav1 wrky6-2* double mutant had much lower cotyledon-greening percentages compared with wild-type plants (Fig 10E). The expression of *ABIs* was also tested, and the qRT-PCR results showed that the transcript levels of *ABI3*, *ABI4*, and *ABI5* were elevated in the *rav1 wrky6-2* double mutant, similar to that in the *rav1* mutant, typically under ABA treatment (Fig 10F).

Taken together, these results demonstrate that disruption of *RAV1* abolishes the ABA-insensitivity of the *wrky6-2* mutant.

### WRKY6 can not directly regulate the expression of *ABI3*, *ABI4* and *ABI5*

**Fig 11:**
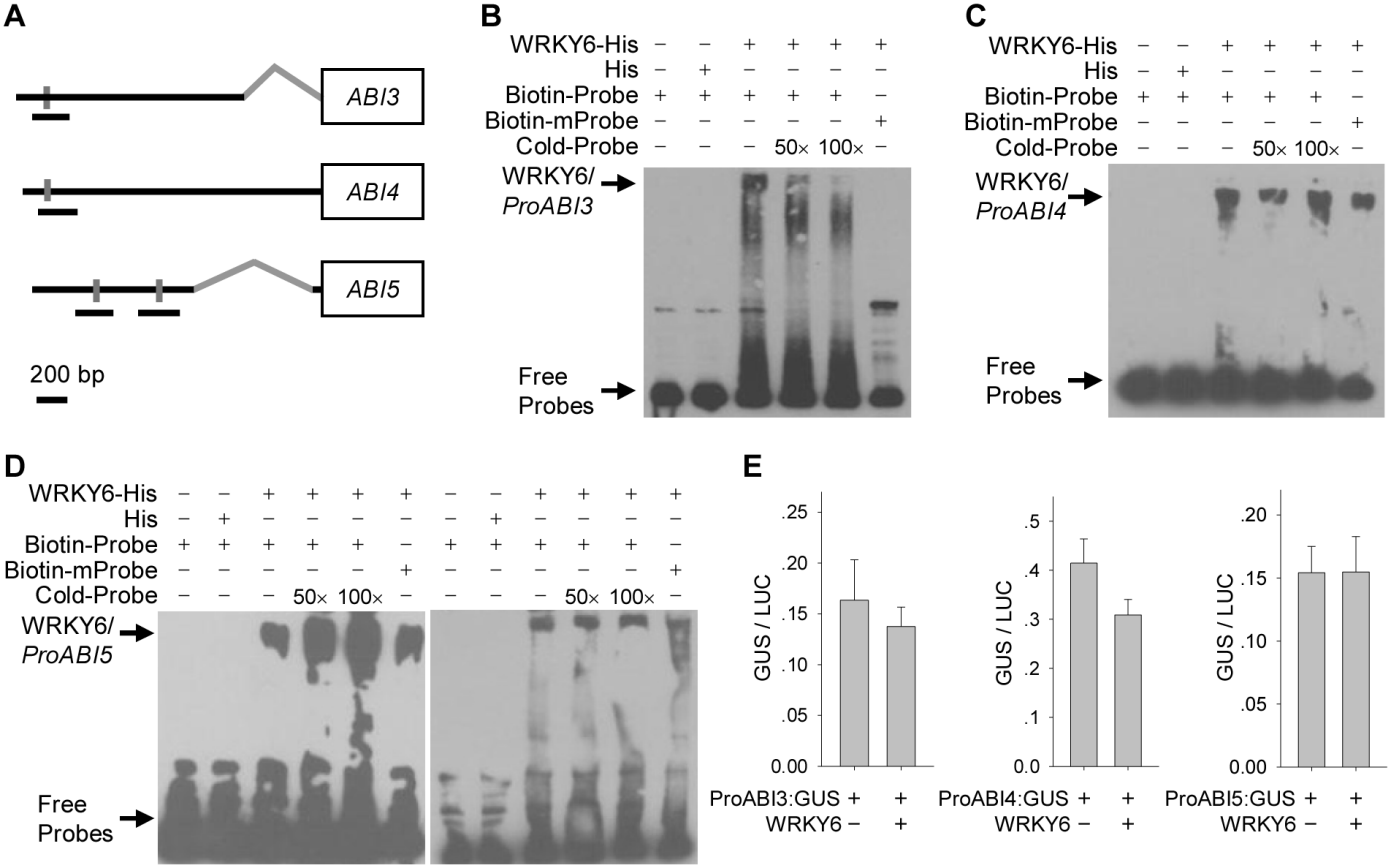
WRKY6 can not directly regulate the expression of *ABI3*, *ABI4* and *ABI5*. A, Diagrams of *ABI3*, *ABI4* and *ABI5* promoters showing relative positions of W-box motifs (gray lines). Relative positions and sizes of different PCR-amplified fragments are indicated by black lines under the W boxes. The gray bent lines indicate the introns within the 5’UTR of *ABI3* and *ABI5*. B-D, EMSA of WRKY6 binding to promoters of *ABI3*, *ABI4* and *ABI5 in vitro*. Each biotin-labeled DNA probe was incubated with WRKY6-His protein. The mutation probes have the mutated W-box (TTGACC was replaced by TACGTC). E, Transient overexpression of *WRKY6* fused to *ProABIs*:*GUS* in *Nicotiana benthamiana* leaves. Data are shown as mean ± SE (n = 6).

There were one or two W boxes within the promoters of *ABI3*, *ABI4* and *ABI5* (Fig 11A), and the expression of *ABI3*, *ABI4* and *ABI5* was elevated in the *WRKY6*-overexpressing lines and repressed in the *wrky6* mutants (Fig 2). It is hypothesized that WRKY6 directly regulates the expression of *ABI3*, *ABI4* and *ABI5*. Then the EMSA experiment was conducted, and the results showed that WRKY6 can bind to the W-box within the *ABI3* promoter *in vitro* (Fig 11B). Although the super-shifted WRKY6-ProABI4 and WRKY6-ProABI5 complexes were detected, these bindings were not reduced by adding the unlabeled competitors, or not missing with the mutation probe with the mutated W-box (TTGACC was changed to TACGTC) (Fig 11C and 11D), indicating that WRKY6 can not bind to the promoters of *ABI4* and *ABI5 in vitro*. To further test the function of WRKY6 in regulation of *ABI3*, *ABI4*, and *ABI5* expression, the transient expression experiment in tobacco leaves was performed. Although WRKY6 can bind to the *ABI3* promoter *in vitro*, WRKY6 can not regulate *ABI3* expression in tobacco leaves (Fig 11E). And WRKY6 can not regulate the expression of *ABI4* and *ABI5* in tobacco leaves (Fig 11E). All these data indicate that WRKY6 can not directly regulate the expression of *ABI3*, *ABI4*, and *ABI5*.

## Discussion

### WRKY6 is an important regulator in ABA signaling during seed germination and early seedling development

*Arabidopsis* WRKY6 is a WRKY transcription factor [25]. In this study, we demonstrated that *Arabidopsis* WRKY6 played important roles in ABA signaling during seed germination and early seedling development. When germinated and grown on MS medium containing ABA, the *wrky6* mutants were ABA-insensitive while *WRKY6*-overexpressing lines were ABA-hypersensitive compared with wild-type plants (Fig 1). As a WRKY transcription factor, WRKY6 is localized in the nucleus and has a DNA-binding domain (WRKY domain) [25]. One reason for the ABA-response phenotypes is that WRKY6 regulated the expression of ABA-response genes. The AREB/ABFs are bZIP-type transcription factors, which recognize the ABA-responsive elements (ABRE) in the promoters of ABA-inducible genes [27], and the expression of *AREB1/ABF2*, *AREB2/ABF4* and *ABF3* is induced by dehydration, high salinity and ABA treatment in vegetative tissues [33]. The expression of *ABF2* and *ABF3* was induced by exogenous ABA, and this inducement was obviously repressed in the *wrky6* mutants (Fig 3), and the cotyledon-greening percentages of *wrky6-1* and *wrky6-2* were much higher than wild-type plants (Fig 1I), indicating that WRKY6 played a role in response to ABA signaling during post-germination growth partially by regulating expression of *ABF2* and *ABF3*. Further promoter sequence analysis results showed that there was no W box (TTGACC/T) within the 2-kb promoters of *ABF1*, *ABF2* and *ABF3*, indicating that WRKY6 can not directly regulate the expression of *ABF1*, *ABF2* and *ABF3*.

The transcription factors ABI3, ABI4, and ABI5 are well known positive regulators of ABA signaling during seed germination [10–12]. The qRT-PCR results showed that the transcript levels of *ABI3*, *ABI4*, and *ABI5*—typically the expression of *ABI3* and *ABI4*—were repressed in *wrky6* mutants (*wrky6-1* and *wrky6-2*) and elevated in *WRKY6*-overexpressing lines (Figs 2 and 3), suggesting that WRKY6 modulated the expression of *ABI3*, *ABI4*, and *ABI5*. The qRT-PCR results also showed that *RAV1* expression was significantly induced in *wrky6* mutants (*wrky6-1* and *wrky6-2*) and repressed in *WRKY6*-overexpressing lines (*35S*:*WRKY6-5* and *35S*:*WRKY6-9*) (Figs 2 and 3). Our previous work showed that the *Arabidopsis* RAV1 transcription factor negatively regulated the expression of *ABI3*, *ABI4*, and *ABI5* [30], suggesting that WRKY6 modulates the expression of *ABI3*, *ABI4*, and *ABI5* by negatively regulating *RAV1* expression. The EMSA and ChIP analyses showed that WRKY6 could bind to the *RAV1* promoter *in vitro* and *in vivo* (Fig 5), demonstrating that WRKY6 negatively regulated *RAV1* expression by binding to the *RAV1* promoter.

Usually, WRKY transcription factors contain the conserved WRKY domain and bind to the W box(es) within their target genes’ promoters [18–19]. Interestingly, the genes *ABI3*, *ABI4*, and *ABI5* contain several W boxes in their promoters [20, 23], and the expression of *ABI3*, *ABI4*, and *ABI5* was enhanced in *WRKY6*-overexpressing lines and repressed in *wrky6* mutants (Figs 2 and 3). Previous reports showed that WRKY40 directly represses *ABI5* expression [20], and WRKY41 directly regulates *ABI3* expression [23]. Thus we investigated whether WRKY6 directly regulated the expression of *ABI3*, *ABI4*, and *ABI5*, and whether the ABA-response phenotypes of *35S*:*WRKY6* and *wrky6* mutants were due to the direct regulation of WRKY6 on *ABI3*, *ABI4*, and *ABI5*. The phenotype of *35S*:*WRKY6-9/RAV1 OE2* was first tested. When grown on MS medium containing ABA, the *WRKY6*-overexpressing line (*35S*:*WRKY6-9*) showed an ABA-hypersensitive phenotype, whereas overexpression of *RAV1* in *35S*:*WRKY6-9* (*35S*:*WRKY6-9/RAV1 OE2*) repressed the ABA-hypersensitivity of *35S*:*WRKY6-9* (Fig 6), indicating that its ABA-hypersensitivity was mainly due to repression of *RAV1* by WRKY6. And the transcript levels of *ABI3*, *ABI4* and *ABI5* in *wrky6-2 RAV1 OE* were lower than those in *wrky6-2* mutant, similar to those in *RAV1 OE2* (Fig 8), indicating that the expression of *ABI3*, *ABI4* and *ABI5* was regulated by RAV1, not by WRKY6. Then the *wrky6 RAV1-U* and *rav1 wrky6-2* double mutant was generated. When grown on MS medium containing ABA, the *wrky6-2* mutant showed an ABA-insensitive phenotype, and the repression or disruption of *RAV1* in the *wrky6-2* mutant (i.e. *wrky6-2 RAV1-U* and *rav1 wrky6-2*) abolished the ABA-insensitivity of *wrky6-2* (Figs 9 and 10), indicating that the ABA-insensitivity of *wrky6-2* was mainly due to the disruption of the regulation by WRKY6 of *RAV1*, and *RAV1* was epistatic to *WRKY6*. The EMSA results showed that WRKY6 could not bind to the promoters of *ABI4* (Fig 11C) and *ABI5* (Fig 11D), indicating that WRKY6 could not directly regulate the expression of *ABI4* and *ABI5*. WRKY6 can bind to the *ABI3* promoter *in vitro* (Fig 11B), whereas WRKY6 can not modulate the *ABI3* expression in plants (Fig 11E), indicating that WRKY6 also can not directly regulate *ABI3* expression. These data demonstrate that WRKY6 acted as a positive regulator mainly via direct regulation of *RAV1* expression.

The 2-kb promoter sequences of *SnRK2s* and *Ems* were also analyzed, and the results showed that there was no W box in *SnRK2*.*2* promoter, one in *SnRK2*.*6* and *Em1* promoters, two in *SnRK2*.*3* promoter and three in *Em6* promoter. And the transcript levels of the *SnRK2s* and *Ems* were elevated in the *WRKY6*-overexpressing lines (Fig 2) and repressed in the *wrky6* mutants under ABA treatment (Fig 3), indicating that WRKY6 transcription factor may directly regulate the expression of *SnRK2*.*3*, *SnRK2*.*6*, *Em1* and *Em6*.

**Fig 12:**
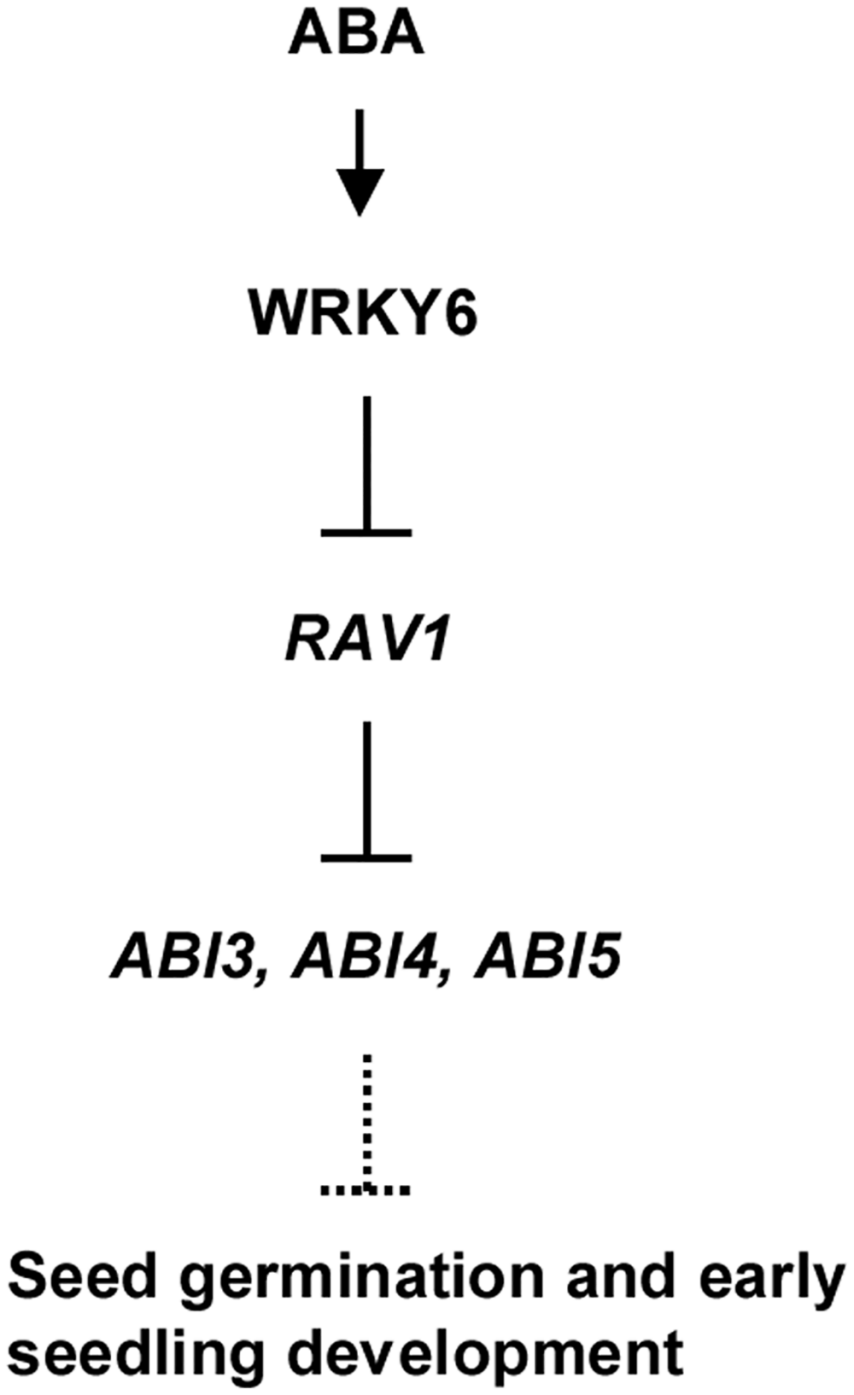
Hypothetical model of WRKY6/RAV1/ABIs-regulatory pathway in plant responses to ABA signaling during seed germination and early seedling development. ABA induces the activity of WRKY6, and WRKY6 binds to the *RAV1* promoter to repress *RAV1* expression. RAV1 directly represses the expression of *ABI3*, *ABI4* and *ABI5*, which promote seed germination and early seedling development.

In summary, our data show that the *Arabidopsis* WRKY6 transcription factor plays important roles in ABA signaling (Fig 12). The *WRKY6* expression is repressed during seed germination and early seedling development, and induced by exogenous ABA. WRKY6 transcription factor acts in the ABA signal transduction pathway predominantly by directly down-regulating *RAV1* expression; RAV1 mediates seed germination and early seedling development by directly down-regulating expression of *ABI3*, *ABI4* and *ABI5*.

### WRKY6 plays important roles in plant development and stress response

The *WRKY6* gene, encoding a WRKY transcription factor, is expressed in all tissues [25], suggesting that WRKY6 plays widespread roles during different phases of plant development. The *WRKY6* transcript is present in roots, shoots, flowers, siliques and senescent leaves, with the highest transcript level of *WRKY6* in senescent leaves [25]. Overexpression of *WRKY6* results in dwarfed *Arabidopsis* with partly necrotic leaves, early flowering and a reduction in their apical dominance [26]. Interestingly, overexpressing *RAV1* caused a retardation of rosette leaf development, and underexpression of *RAV1* caused an earlier flowering phenotype [32]. Recently, *Arabidopsis* RAV1 was reported to positively regulate leaf senescence, and overexpression of *RAV1* caused premature leaf senescence [34]. The data in the present study showed that WRKY6 directly repressed *RAV1* expression. The data suggested that the WRKY6-RAV1 regulatory pathway was involved in leaf senescence and flowering.

In addition to modulating leaf senescence and flowering, the expression of *WRKY6* was repressed during seed germination and early seedling development, and obviously induced by exogenous ABA (Fig 1A–1C). When grown on MS medium with ABA, the *wrky6* mutants showed ABA-insensitive phenotypes while the *WRKY6*-overexpressing lines were ABA-hypersensitive (Fig 1E). WRKY6 could bind to the *RAV1* promoter to repress *RAV1* expression (Fig 5). Further genetic results showed that *RAV1* was the main target gene of WRKY6 during seed germination and early seedling development (Figs 6–10). These data provide evidence of the major role of WRKY6 during seed germination and early seedling development.

WRKY6 is also involved in controlling processes related to pathogen defense [25–26]. WRKY6 positively influences the promoter activity of the pathogen defense-associated *PR1* gene, most likely involving NPR1 function [26]. In addition to abiotic stress, WRKY6 has also been reported to be involved in biotic stress responses. WRKY6 is a negative regulator in phosphate translocation [35]. When grown on inorganic phosphorus (Pi)-sufficient condition, WRKY6 represses *PHO1* expression and reduces Pi translocation from roots to shoots. During Pi starvation, the WRKY6 protein is degraded and the repression of *PHO1* by WRKY6 is abolished [35]. The expression of *WRKY6* is induced by boron (B) deficiency, and *wrky6* mutants showed growth defects compared with wild-type plants under B deficient condition [36]. Recently, *WRKY6* was reported to be induced by arsenate stress, and the WRKY6 mediated the expression of a phosphate transporter gene and restricted arsenate-induced transposon activation [37]. Taken together, the WRKY6 transcription factor plays important roles in plant development and biotic and abiotic stress responses.

## Materials and Methods

### Plant materials and growth conditions

The wild-type plant used in this study was *A*. *thaliana* Col-0. The *WRKY6*-overexpressing lines (*35S*:*WRKY6-5* and *35S*:*WRKY6-9*), the *wrky6-1* mutant, the *RAV1*-overexpressing line (*RAV1 OE2*) and the *RAV1*-underexpressing line (*RAV1-U*) were described previously [26, 30, 32]. The *WRKY6* T-DNA insertion line Salk_012997, named *wrky6-2* in the present study, was ordered from the ABRC. For the seed germination assay, seeds were surfaced sterilized and kept at 4°C for 72 h in darkness before germination. About 300 seeds of each genotype were sown on the same plate containing MS medium [with 3% (w/v) sucrose] with 0, 0.5 and 2 μM ABA, and were kept at 22°C under constant illumination of 60 μmol·m^−2^·s^−1^. Germination was defined as an obvious emergence of the radicle through the seed coat. The seed germination percentages were evaluated daily during the germination test.

### qRT-PCR assay

For qRT-PCR analysis, total RNA of seedlings and seeds was extracted with Trizol reagent (Invitrogen) and RNeasy Plant Mini kit (Bioteke), separately. The total RNA (8 μg) was treated with DNase I (RNase Free) (Takara) to eliminate genomic DNA contamination. Then the cDNA was synthesized from the treated total RNA (4 μg) by SuperScript II Reverse Transcriptase (Invitrogen) using Radom Hexamer Primers (Promega). 40 ng cDNA (except *RAB18*, with 80 ng cDNA) and 50 nM each primer were used for each quantitative PCR reaction, which was performed by using the Power SYBR Green PCR Master Mix (Life Technologies) on a 7500 Real Time PCR System machine (Life Technologies) following the manufacturer’s protocols. The thermal treatment was 10 min at 95°C, then 40 cycles of 15 s at 95°C, 1 min at 60°C. Amplification was followed by a melt curve analysis. The 2^-ΔΔCt^ method was used for relative quantification [38]. *Actin2/8* expression was used as an internal control. The statistical significance was evaluated by Paired t-test analysis. The primers used are listed in S1 Table.

### Transient expression assay in *Nicotiana benthamiana*

The transient GUS expression assay was performed as described previously [35]. The *ProRAV1*:*GUS* and *Super*:*WRKY6* constructs were described previously [30, 35]. To construct *RroABI3*, *ProABI4* and *ProABI5*, the ∼2kb promoters of *ABI3*, *ABI4* and *ABI5* were cloned into the *pCAMBIA1381* vector. The primer sequences used are listed in S1 Table. For each infiltration sample, *Super*:*LUC* was added as an internal control. The GUS and LUC activities of the infiltrated leaves were quantitatively determined, and the GUS/LUC ratio was used to quantify the promoter activity.

### Protein expression and EMSA experiment

The coding sequence of *WRKY6* was amplified and cloned into the *pET30a* vector. The primer sequences used are listed in S1 Table. The recombinant plasmid was introduced to *E*. *coli* strain BL21. *E*. *coli* cells were induced with 0.2 mM IPTG overnight at 18°C and collected by centrifugation. The WRKY6-His protein was purified using Ni-Sepharose 6 Fast Flow (GE Healthcare), and the protein concentration was determined by Bio-Rad protein assay. The pET30a vector was also introduced into *E*. *coli* strain BL21, and a protein with His tag was purified. This purified protein was named His protein, and used as a control in EMSA experiment.

For EMSA assays, the fragment of the promoters were obtained by PCR using biotin-labeled or -unlabeled primers (see S1 Table). Biotin-unlabeled fragments of the same sequences were used as competitors. The reaction mixture (20 μL) for EMSA contained 0.5 μg purified protein, 1 μL 50 μg/mL biotin-labeled annealed oligonucleotide, 2 μL 10×binding buffer (100 mM Tris, 500 mM KCl, and 10 mM DTT, pH 7.5), 1 μL 1% Nonidet P-40, 0.5 μL 1 mg/mL poly (dI-dC), and ultrapure water. The reactions were incubated at 22°C for 30 min. The reactions were fractionated on a 5% native polyacrylamide gel in 0.5 ×TBE buffer. The detection of biotin-labeled DNA by chemiluminescence was performed using a LightShift Chemiluminescent EMSA Kit (Pierce) following the manufacturer’s protocol.

### ChIP-qPCR assay

The ChIP experiment was performed as described previously [30, 35]. For the ChIP assay, 1 g of 7-d-old seedlings grown on MS medium was transferred to MS solution with or without 100 μM ABA for 3 h, then harvested and cross-linked by 1% formaldehyde for 10 min, and then the purified cross-linked nuclei were resuspended in 4 mL lysis buffer. Following sonication, 1 mL lysis buffer with nuclei was used for each immunoprecipitation (IP). The anti-WRKY6 antibody (AS111778; Agrisera, http://www.agrisera.com/) was used to immunoprecipitate DNA/protein complexes from the chromatin preparation. IP DNA was dissolved in 25 μL TE buffer, and 1 μL IP DNA was analyzed by qPCR using the primers listed in S1 Table. As a control, ‘input’ DNA was isolated from 50 μL lysis buffer with nuclei without the IP step. The input DNA was suspended in 25 μL TE buffer and 1 μL input DNA was analyzed by qPCR. The ratio of IP DNA over the input was presented as the percentage of input (IP %). An *Actin* fragment (ACTIN) was amplified as control. At least three independent experiments were performed with similar results. Data are mean values of three replicates ± standard error (SE) from one experiment.

### Generation of *rav1* mutant and *rav1 wrky6-2* double mutant using CRISPR/Cas9 technology

A pair of closely located sgRNA targets (C1: GATGAGAGTACTACAAGTAC and C2: ACGGCGTAGAAGCTGAATCT) in *RAV1* gene was selected and cloned into the pHEE2A-TRI vector as described [39]. Then the CRISPR construct was transformed into wild-type *Arabidopsis* and the *wrky6-2* mutant to obtain *rav1* mutant and *rav1 wrky6-2* double mutant, separately. The homozygous *rav1* mutant and *rav1 wrky6-2* double mutant were identified by sequencing.

### Accession numbers

Sequence data for the *Arabidopsis* genes described in this study can be found in the Arabidopsis Genome Initiative or GenBank/EMBL databases under the following accession numbers, At1g62300 for WRKY6, At1g49720 for ABF1, At1g45249 for ABF2, At4g34000 for ABF3, At3g50500 for SnRK2.2, At5g66880 for SnRK2.3, At4g33950 for SnRK2.6, At3g24650 for ABI3, At2g40220 for ABI4, At2g36270 for ABI5, At1g13260 for RAV1, At3g51810 for Em1, and At2g40170 for Em6.

## Supporting Information

S1 Fig Phenotypic comparison. (PDF)

S1 Table Primer sequences used in this study. (PDF)
