# Correction: *Arabidopsis* WRKY6 Transcription Factor Acts as a Positive Regulator of Abscisic Acid Signaling during Seed Germination and Early Seedling Development

**DOI:** 10.1371/journal.pgen.1008032

**Published:** 2019-03-06

**Authors:** Yun Huang, Cui-Zhu Feng, Qing Ye, Wei-Hua Wu, Yi-Fang Chen

There are errors contained within the caption for Fig 1 and errors in the designation of primer sequences used in S1 Table. Please find a corrected version of Fig 1 legend and S1 Table here.

**Fig 1:**
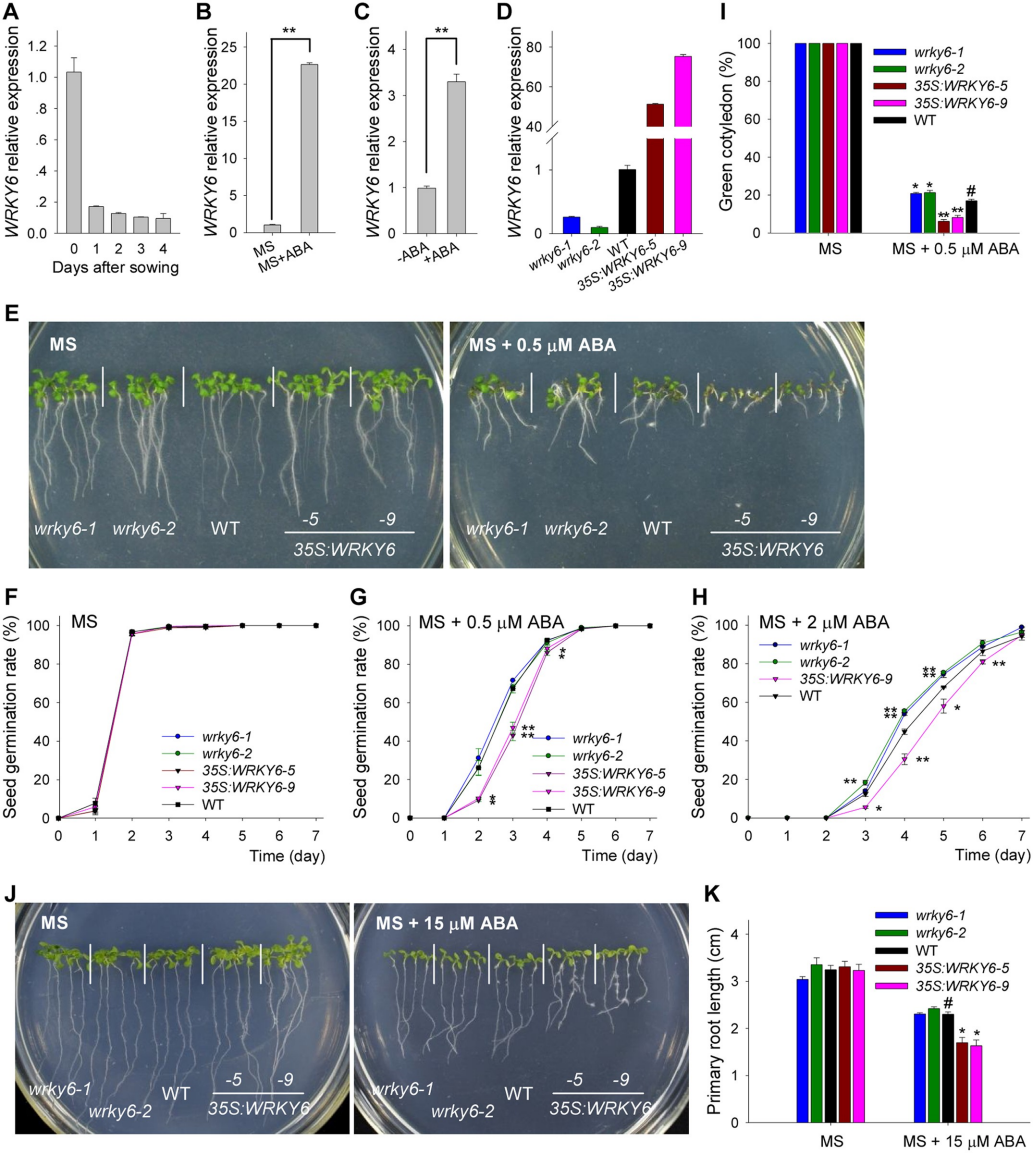
ABA-sensitivity of *wrky6* mutants and *WRKY6*-overexpressing lines. A, Expression of *WRKY6* was analyzed by qRT-PCR in wild-type plants (WT) during seed germination and early seedling development. The wild-type imbibed seeds were germinated and grown on MS medium, and then the plants were harvested at the indicated time. Data are shown as mean ± SE (n = 3). B, qRT-PCR analysis of *WRKY6* expression in response to exogenous ABA. Wild-type imbibed seeds were germinated on MS medium (MS) or MS medium with 0.5 μM ABA (MS+ABA) for 1 d, and then the seeds were harvested. Data are shown as mean ± SE (n = 3). C, qRT-PCR analysis of *WRKY6* expression in 7-d-old wild-type seedlings treated with or without 100 μM ABA for 3 h. Data are shown as mean ± SE (n = 3). D, Expression of *WRKY6* was analyzed by qRT-PCR in the *wrky6* mutants (*wrky6-1* and *wrky6-2*) and *WRKY6*-overexpressing lines (*35S*:*WRKY6-5* and *35S*:*WRKY6-9*). Data are shown as mean ± SE (n = 3). E, Phenotypic comparison. Imbibed seeds were transferred to MS or MS + 0.5 μM ABA medium and grown for 10 d. F-H, Seed germination assay. Imbibed seeds were transferred to MS (F), MS medium containing 0.5 μM ABA (G) or 2 μM ABA (H), and then the seed germination rates were calculated at the indicated time. Data are shown as mean ± SE (n = 3). More than 300 seeds were measured in each replicate. I, Cotyledon-greening analysis. Imbibed seeds were transferred to MS or MS + 0.5 μM ABA medium for 7 d before determining cotyledon-greening percentages. Data are shown as mean ± SE (n = 3). More than 300 seeds were measured in each replicate. J-K, Primary root length measurement with and without ABA addition. The 4-d-old seedlings were transferred to MS or MS + 15 μM ABA medium for 7 d, and then the photos were taken and the primary root length was measured. Asterisks in G, H, I and K indicate statistically significant differences compared with wild-type plants: *, *P <* 0.05; **, *P <* 0.01. Wild-type plant (WT) was used as a control (#).

## Supporting information

S1 Table Primer sequences used in this study. (PPTX)
