# Supplementary material for: Arabidopsis WRKY6 Transcription Factor Acts as a Positive Regulator of Abscisic Acid Signaling during Seed Germination and Early Seedling Development
Source: PLoS Genet. 2016 Feb 1;12(2):e1005833. doi: 10.1371/journal.pgen.1005833 (PMC4734665; doi:10.1371/journal.pgen.1005833)
Supplement: S1 Fig — (PDF) [file pgen.1005833.s001.pdf]

Supplemental Figure S1

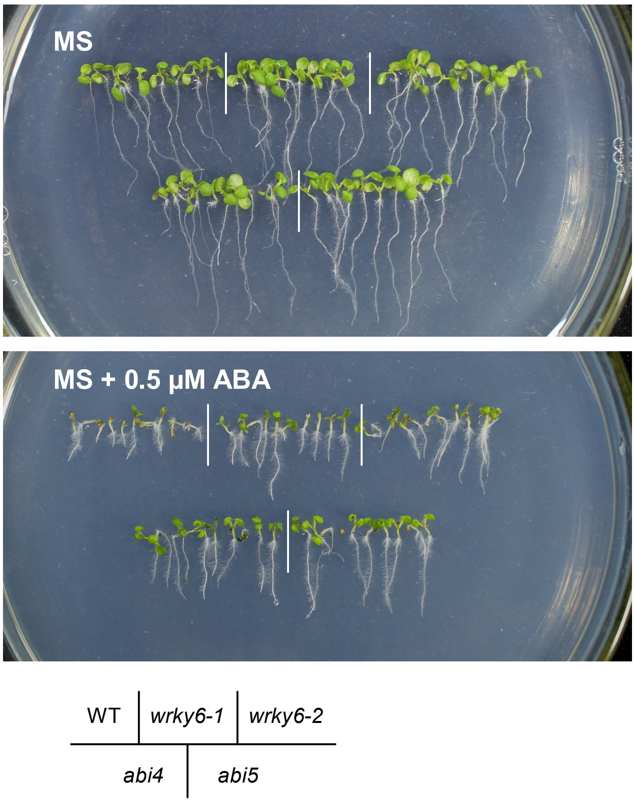

Fig. S1. Phenotypic comparison.
Imbibed seeds were transferred to MS or MS + 0.5 μM ABA medium and grown for 8 d.
