# Supplementary material for: Arabidopsis WRKY6 Transcription Factor Acts as a Positive Regulator of Abscisic Acid Signaling during Seed Germination and Early Seedling Development
Source: PLoS Genet. 2016 Feb 1;12(2):e1005833. doi: 10.1371/journal.pgen.1005833 (PMC4734665; doi:10.1371/journal.pgen.1005833)
Supplement: S1 Table — (PDF) [file pgen.1005833.s002.pdf]

Supplemental Table 1. Primer sequences used in this study.

Primer Name	Forward Primer (5' → 3')	Reverse Primer (5' → 3')
Primers for qRT-PCR		
Actin2/8	ACGGTAACATTGTGCTCAGTGGTG	CTTGGAGATCCACATCTGCTGGA
WRKY6	GAGGCCGAGGATGTGTCAAA	GAGGCCGAGGATGTGTCAAA
RAV1	GAGGCCGAGGATGTGTCAAA	GAGGCCGAGGATGTGTCAAA
ABI3	TCCATTAGACAGCAGTCAAGGTTT	GGTGTCAAAGAACTCGTTGCTATC
ABI4	GGGCAGGAACAAGGAGGAAG TG	ACGGCGGTGGATGAGTTATTGAT
ABI5	CAATAAGAGAGGGATAGCGAACGAG	CAATAAGAGAGGGATAGCGAACGAG
Em1	TAGGGCACGAGGGTTATCAG	CGCTCTCCACCAGATTTTTC
Em6	GCAAACTCGAAAGGAGCAGT	TCTCGACTCCTTCCTCCTCA
ABF1	TCAACAACTTAGGCGGCGATAC	GCAACCGAAGATGTAGTAGT
ABF2	TTGGGGAATGAGCCACCAGGAG	GACCCAAAATCTTTCCCTACAC
ABF3	CTTTGTTGATGGTGTGAGTGAG	GTGTTTCCACTATTACCATTGC
SnRK2.2	CCGGAGATCACATCCGATAA	ATGAAATCATCGAGGCAACG
SnRK2.3	CAAATTTGCCATCGTGACCT	GCAGTACCTCTGGAGCGATG
SnRK2.6	AGATCCCGAGGAACCAAAGA	CTCTTTGCAGGGTCAGCAAC
Primers for ChIP and EMSA		
Actin	CCGGTATTGTGCTCGATTCTG	TTCCCGTTCTGCGGTAGTGG
RAV1 P1	GCCCACAACATATTCATTTC	ATATTTGTAAGAATGCGTGTTT
RAV1 P2	GATAAGATCGTAAGCTATCCAC	GTTTCTCATAAGTGGGTG
ABI3	ATTTGGTTTGAAGAACATGCC	GGAAAATTCCTATTTTCCCG
ABI4	GGTGTAGTATCCAAAATCTAAATCTATCG	CTGGAGTAGTACTTGTTAAATTTTGGAG
ABI5 -1	TAGCCGAAGTCACACGTGT	AGGAGGCGGTAAAAGAGAGAT
ABI5-2	TCTCGACTTTGATTTGTTTTAA	ACATATTGGTTCATGATTTG
Primers for protein expression in *E. coli.*		
WRKY6-His	GGCTTAAUGACAGAGGATGGTCTGGTCTCAC	GGTTTAAUTTGATTTTTGTTGTTTCCTTCGC
For Genotyping		
wrky6-2 LP	GAACGTATTAGCCAATCACGC	
wrky6-2 RP	TGTGGACGTGTCATAATTTGG	
LBb1.3	ATTTTGCCGATTTCGGAAC	
For ProABIs:GUS construct		
ProABI3	GAATTCACTAAAATTTTGATGTGTTAA	CTGCAGATCGTTGAAGTGGAAAT
ProABI4	GAATTCAAGCGTACATAAATATCTACACTATAACTCCTC	GTCGACAGATGAAGAAGAAGAAGAAGAAGAAGA
ProABI5	GGATCCTCTACTTTCACCAGCTAGAA	CTGCAGATTTAACAACTGCATCATATAC
