# Supplementary material for: Correction: Arabidopsis WRKY6 Transcription Factor Acts as a Positive Regulator of Abscisic Acid Signaling during Seed Germination and Early Seedling Development
Source: PLoS Genet. 2019 Mar 6;15(3):e1008032. doi: 10.1371/journal.pgen.1008032 (PMC6402642; doi:10.1371/journal.pgen.1008032)
Supplement: S1 Table — (PPTX) [file pgen.1008032.s001.pptx]

## Slide 1
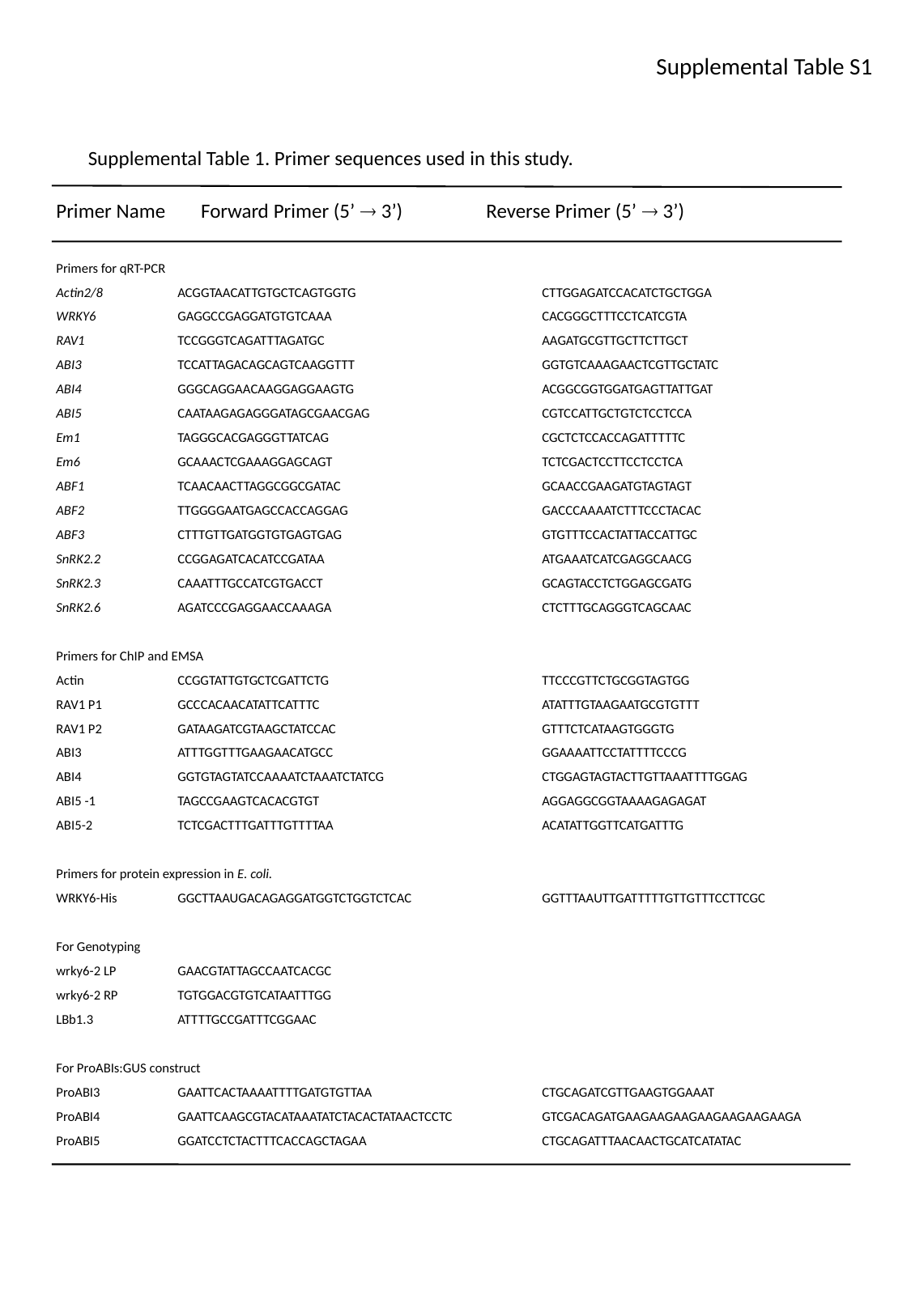

Supplemental Table S1
Supplemental Table 1. Primer sequences used in this study.
Primer Name	 Forward Primer (5’  3’) Reverse Primer (5’  3’)
Primers for qRT-PCR
Actin2/8	ACGGTAACATTGTGCTCAGTGGTG		CTTGGAGATCCACATCTGCTGGA
WRKY6	GAGGCCGAGGATGTGTCAAA		CACGGGCTTTCCTCATCGTA
RAV1	TCCGGGTCAGATTTAGATGC 		AAGATGCGTTGCTTCTTGCT
ABI3	TCCATTAGACAGCAGTCAAGGTTT		GGTGTCAAAGAACTCGTTGCTATC
ABI4	GGGCAGGAACAAGGAGGAAGTG		ACGGCGGTGGATGAGTTATTGAT
ABI5	CAATAAGAGAGGGATAGCGAACGAG		CGTCCATTGCTGTCTCCTCCA
Em1	TAGGGCACGAGGGTTATCAG		CGCTCTCCACCAGATTTTTC
Em6	GCAAACTCGAAAGGAGCAGT		TCTCGACTCCTTCCTCCTCA
ABF1	TCAACAACTTAGGCGGCGATAC		GCAACCGAAGATGTAGTAGT
ABF2	TTGGGGAATGAGCCACCAGGAG		GACCCAAAATCTTTCCCTACAC
ABF3	CTTTGTTGATGGTGTGAGTGAG		GTGTTTCCACTATTACCATTGC
SnRK2.2	CCGGAGATCACATCCGATAA		ATGAAATCATCGAGGCAACG
SnRK2.3	CAAATTTGCCATCGTGACCT		GCAGTACCTCTGGAGCGATG
SnRK2.6	AGATCCCGAGGAACCAAAGA		CTCTTTGCAGGGTCAGCAAC
Primers for ChIP and EMSA
Actin	CCGGTATTGTGCTCGATTCTG		TTCCCGTTCTGCGGTAGTGG
RAV1 P1	GCCCACAACATATTCATTTC		ATATTTGTAAGAATGCGTGTTT
RAV1 P2	GATAAGATCGTAAGCTATCCAC		GTTTCTCATAAGTGGGTG
ABI3	ATTTGGTTTGAAGAACATGCC		GGAAAATTCCTATTTTCCCG
ABI4	GGTGTAGTATCCAAAATCTAAATCTATCG		CTGGAGTAGTACTTGTTAAATTTTGGAG
ABI5 -1	TAGCCGAAGTCACACGTGT		AGGAGGCGGTAAAAGAGAGAT
ABI5-2	TCTCGACTTTGATTTGTTTTAA		ACATATTGGTTCATGATTTG
Primers for protein expression in E. coli.
WRKY6-His	GGCTTAAUGACAGAGGATGGTCTGGTCTCAC		GGTTTAAUTTGATTTTTGTTGTTTCCTTCGC
For Genotyping
wrky6-2 LP	GAACGTATTAGCCAATCACGC
wrky6-2 RP	TGTGGACGTGTCATAATTTGG
LBb1.3	ATTTTGCCGATTTCGGAAC
For ProABIs:GUS construct
ProABI3	GAATTCACTAAAATTTTGATGTGTTAA		CTGCAGATCGTTGAAGTGGAAAT
ProABI4	GAATTCAAGCGTACATAAATATCTACACTATAACTCCTC	GTCGACAGATGAAGAAGAAGAAGAAGAAGAAGA
ProABI5	GGATCCTCTACTTTCACCAGCTAGAA		CTGCAGATTTAACAACTGCATCATATAC
